# “The First Thousand Days” Define a Fetal/Neonatal Neurology Program

**DOI:** 10.3389/fped.2021.683138

**Published:** 2021-08-02

**Authors:** Mark S. Scher

**Affiliations:** Division of Pediatric Neurology, Department of Pediatrics, Fetal/Neonatal Neurology Program, Emeritus Scholar Tenured Full Professor in Pediatrics and Neurology, Case Western Reserve University School of Medicine, Cleveland, OH, United States

**Keywords:** the first thousand days, developmental neuroplasticity, fetal/neonatal neurology, maternal/placental/fetal triad, neonatal neurocritical care, ontogenetic adaptation, developmental origins and life-course theories, pediatric neurology

## Abstract

Gene–environment interactions begin at conception to influence maternal/placental/fetal triads, neonates, and children with short- and long-term effects on brain development. Life-long developmental neuroplasticity more likely results during critical/sensitive periods of brain maturation over these first 1,000 days. A fetal/neonatal program (FNNP) applying this perspective better identifies trimester-specific mechanisms affecting the maternal/placental/fetal (MPF) triad, expressed as brain malformations and destructive lesions. Maladaptive MPF triad interactions impair progenitor neuronal/glial populations within transient embryonic/fetal brain structures by processes such as maternal immune activation. Destructive fetal brain lesions later in pregnancy result from ischemic placental syndromes associated with the great obstetrical syndromes. Trimester-specific MPF triad diseases may negatively impact labor and delivery outcomes. Neonatal neurocritical care addresses the symptomatic minority who express the great neonatal neurological syndromes: encephalopathy, seizures, stroke, and encephalopathy of prematurity. The asymptomatic majority present with neurologic disorders before 2 years of age without prior detection. The developmental principle of ontogenetic adaptation helps guide the diagnostic process during the first 1,000 days to identify more phenotypes using systems-biology analyses. This strategy will foster innovative interdisciplinary diagnostic/therapeutic pathways, educational curricula, and research agenda among multiple FNNP. Effective early-life diagnostic/therapeutic programs will help reduce neurologic disease burden across the lifespan and successive generations.

## Introduction

This review describes clinical pathways integrated into a fetal/neonatal neurology program (FNNP) based on a “first 1,000-day” perspective. FNNPs at two maternal/pediatric medical centers over the past four decades provided the experience to offer this review, bolstered by collaboration with multiple maternal and pediatric subspecialties (1). Diagnostic considerations during antepartum, peripartum, neonatal, and early childhood time-periods were integrated to promote more effective and timelier diagnostic/therapeutic strategies to improve brain health starting early in life. Time-sensitive consultations enhanced short- and long-term outcomes, aided by interdisciplinary input from numerous colleagues. Continuity of pediatric neurology care for patients and their families into adulthood offered longitudinal clinical service, educational and research opportunities. Risks for life-course neurologic burden were communicated to colleagues who then provided care into older ages. These career experiences underscored how integrative prenatal, neonatal, and pediatric care later impacts life-long brain health and recovery from diseases.

## 20th Century Research Emphasized the First 1,000-Day Perspective

Insights regarding the continuity of risk were investigated by 20th century scholarship and public health initiatives. Seminal neonatal neurology research studies were published, notably with many contributions by women across neonatal research disciplines. Advances in neurobehavioral assessments, neurophysiology, neuropathology, and outcomes research highlighted these accomplishments (2, 3).

Post-World War II public health research was conducted in response to concerns for high maternal and pediatric mortality and morbidity. Associations across three trimesters of pregnancy, labor, and delivery events were termed “a continuum of reproductive risk” (4, 5), resulting in neurologic sequelae. The 16-year NIH-funded Collaborative Perinatal Project (CPP) was the American scientific community's prospective research effort to further investigate these epidemiologic findings (6). This birth cohort recruited 58,000 MPF triads, neonates, and children through 8 years of age from 1958–1966. Placental assessments were an important component in the analyses. Neurologic outcomes were described for developmental disorders, epilepsy, and cognitive/behavioral disorders. A “first 1,000-day” perspective, as reflected by these research efforts, was shared across medical disciplines, influencing the growth of the new fields of neonatology and maternal-fetal medicine specialties. Numerous CPP peer-reviewed and secondary source materials led to further research into the 21st century. Open access to these data remains available through the National Archives (6). A transactional model was proposed based in part on this research, to more comprehensively consider reproductive risk and the continuum of care-taking casualty through childhood (7).

The first 1,000-day concept first emphasized the importance of nutritional health for mother and child (8). Multiple factors in addition to nutrition now are recognized that influence complex gene-environment interactions (G × E) from conception, influencing MPF triad, neonatal, and early childhood health. In the 1990s, the Millennium Project proposed maternal and childhood health-care initiatives as overarching public health objectives, suggesting future health policy recommendations (9) with global applications. Sustainable development goals have been more recently revised, citing the birth-cohort and interventional studies from the latter half of the 20th century. A 1,000-day perspective to promote later-life health continues to be emphasized. A FNNP should integrate the complementary theories of developmental origins and life-course science to improve both patient-centered and population health to achieve these sustainable goals. This dual approach will more effectively reduce neurologic burden across the lifespan (1).

## Current Programs Emphasize Neonatal Neurocritical Care

Current NNCCP protocols have focused on peripartum, parturition, and neonatal time-periods when fetal/neonatal neurologic phenotypes are more readily identified based on current diagnostic skills and testing tools. Peripartum fetal surveillance results, neonatal clinical assessment scores, multi-systemic disease monitoring, neuroimaging/EEG protocols, and developmental care strategies applicable to these time-periods comprise the datasets that presently guide clinical care pathways into early childhood (10).

Clinical pathways from conception affecting the MPF triad, however, need to include the NNCCP component into a more comprehensive FNNP to achieve optimal standardization for clinical care, educational, and collaborative research strategies across institutions. Neonates with pre-existing brain malformations or acquired destructive injuries respond less optimally to neonatal neurocritical care and early childhood interventions. Childhood neurologic disorders are also later expressed without requiring or despite NNCCP interventions. Diseases and adversities encountered over the lifespan increase neurologic burden when superimposed on earlier brain injuries or vulnerabilities experienced during the first 1,000 days (11).

Worldwide neonatal neurocritical care programs (NNCCP) continue to expand (12, 13). Sophisticated technologies using enhanced informatics contribute to new diagnostic approaches. Updated secondary sources (14, 15) continue to provide useful summaries and interpretations based on selected peer-reviewed studies. More recently formed professional organizations such as the Newborn Brain Society support clinical service, education, and research efforts, with the present emphasis on neonatal neurocritical care. Multiple NNCCP proposals have even suggested an adult neurointensive care model for service, training, and research program development (16).

NNCCP would be more effective as one of three components of a fetal/neonatal neurology program (FNNP) that assesses the maternal/placental/fetal (MPF) triad from conception until 2 years of life. Developmental neuroscience principles support this first 1,000-day perspective for this proposed FNNP. Life-long effects of developmental neuroplasticity more likely result during critical/sensitive periods of brain maturation, given accelerated experience-dependent effects on brain development during early life. Multiple MPF triad, neonatal and pediatric regulators of plasticity have implications for learning, and other forms of neurologic recovery particularly during the first 1,000 days. Diagnostic/therapeutic interventions require trimester-specific MPF triad evaluations when considering systems-science to explain changes of the developing fetal brain into peripartum and neonatal time-periods. Subsequent care during the first 2 years of life then integrates “the first 1,000-day” perspective into the effects of pediatric/family integrative practice (17), on continued postnatal developmental neuroplasticity. These early-life gene-environment interactions contribute to permanent life-course expressions of brain health or disease throughout childhood into adulthood.

NNCCP strategies presently emphasize diagnostic and therapeutic approaches regarding the great neonatal neurologic syndromes (GNNS) represented by encephalopathy, conditions of prematurity associated with encephalopathy, seizures, and stroke. These disease categories represent the symptomatic minority of MPF triads and livebirths who require neonatal neurointensive care. The majority remains asymptomatic or express less readily detectable diseases during prenatal and neonatal life despite adverse outcomes. Evaluations of MPF triads in a FNNP would more comprehensively address prenatal contributions that influence neurologic sequelae either initially expressed as the GNNS or later as childhood neurologic disorders. Earlier diagnosis would promote timelier and more effective therapeutic interventions.

## Obstacles to Effective Prenatal Medical Care Hamper Life-Course Health

Pediatric specialty consultations for high-risk MPF triads would enhance postnatal care perspectives (18). Prenatal pediatric care, however, is presently challenged by the complexities of MPF triad diseases beyond traditional pediatric training programs to adequately prepare their trainees. Currently, deficiencies in interdisciplinary education persist across multiple specialties including fetal neurology that limit the trainee's career preparation. Inadequate reimbursement for prenatal clinical service remains a disincentive following formal training, discouraging the pediatric neurologist from participating in fetal consultations. Active participation in prenatal consultations would, nonetheless, promote timelier diagnostic input (19) resulting in more effective and anticipatory medical management during pregnancy, neonatal life, and early childhood. Fetal neurology consultations by the pediatric neurologist constitute an important activity within a comprehensive FNNP during the first 1,000 days. Prenatal involvement by the neurologist strengthens diagnostic and therapeutic clinical skills, applicable across the lifespan to improve brain health or mitigate a wide range of diseases, including cerebrovascular, neurodegenerative, and mental health disorders.

Programmatic efforts to include prenatal consultations such as the fetal neurology component of a FNNP presently face multiple challenges. Incomplete clinical/demographic information exists for the majority of maternal-child pairs who receive lower levels of care, followed by outborn deliveries (20, 21) without the availability of advanced resuscitative interventions and neonatal intensive care for unexpected complications. Underdeveloped perinatal regionalization of care result in inadequate access within urban or rural medical deserts (22), even in resource-rich countries. Relevant antepartum information for brain development may not be immediately available to consider when the mother or neonate unexpectantly requires higher levels of care. Trimester-specific MPF triad diseases are often not clinically expressed, or remain undetectable based on the limitations of sensitivity and specificity of present fetal surveillance testing to accurately monitor fetal brain health or disease.

Obstacles to effective prenatal care also exist for the woman, partner, and family as well as the provider. Unanticipated adverse experiences and lifestyle choices constitute formidable barriers to effective prenatal care. Inadequate communication between patient and health-care provider persist because of distrust, institutional racial bias, and socioeconomic barriers (23).

Obstacles encountered during pregnancy also exist after birth for the child and the family who lack adequate health-care access. This is particularly prevalent in resource-poor nations as well as medical deserts in resource-rich nations. Health systems may lack essential obstetrical, neonatal, and pediatric services based on their revenue-limited economies, geographic distances, and cultural/religious practices. Racial/ethnic and socioeconomic factors contribute to medical inequities starting during early childhood, even in resource-rich nations.

Primary-care practitioners may not have full access to relevant clinical information required to optimize preventive or emergent care. Less effective cooperation among competing health systems share incomplete clinical information because of poor internet connectivity or financial priorities. Changes in the family residence and the lack, loss, or reduction of health insurance contribute to less effective pediatric health care in the absence of universal health-care. Life-long negative outcomes of the health of children, families, and communities will consequentially be more likely, and contribute to diseases later in adulthood (24).

Missed interventions during the first 1,000 days to diagnose and therapeutically address fetal/neonatal neurology disorders increase vulnerabilities at older ages when communicable and non-communicable diseases injure the nervous system. Newly diagnosed medical illnesses such as infections and trauma, inadequately managed developmental, and mental disorders such as autism and schizophrenia, and ongoing exposures to adverse environmental conditions such as toxin/pollutants will negatively impact health and quality of life when experienced beyond 2 years of age throughout later childhood and adulthood. These sequelae may also contribute to shortened life expectancy.

Interdependent MPF triad, neonatal, and pediatric/family health conditions, therefore, constitute a continuum of risk with worsening outcome as the nervous system matures throughout childhood into adulthood. Primary nervous system disorders as well as secondary disorders from multi-systemic diseases contribute to a range of adverse neurologic outcomes. Maladaptive responses from experience-dependent plasticity during the first 1,000 days have more permanent negative implications for cognitive potential and degrees of neurologic recovery from diseases experienced at older ages, when encountered during critical/sensitive developmental periods of brain maturation over the first 1,000 days (25).

## The Dual Diagnostic Process Guides Fetal/Neonatal Neurology Consultations

**Figure 1:**
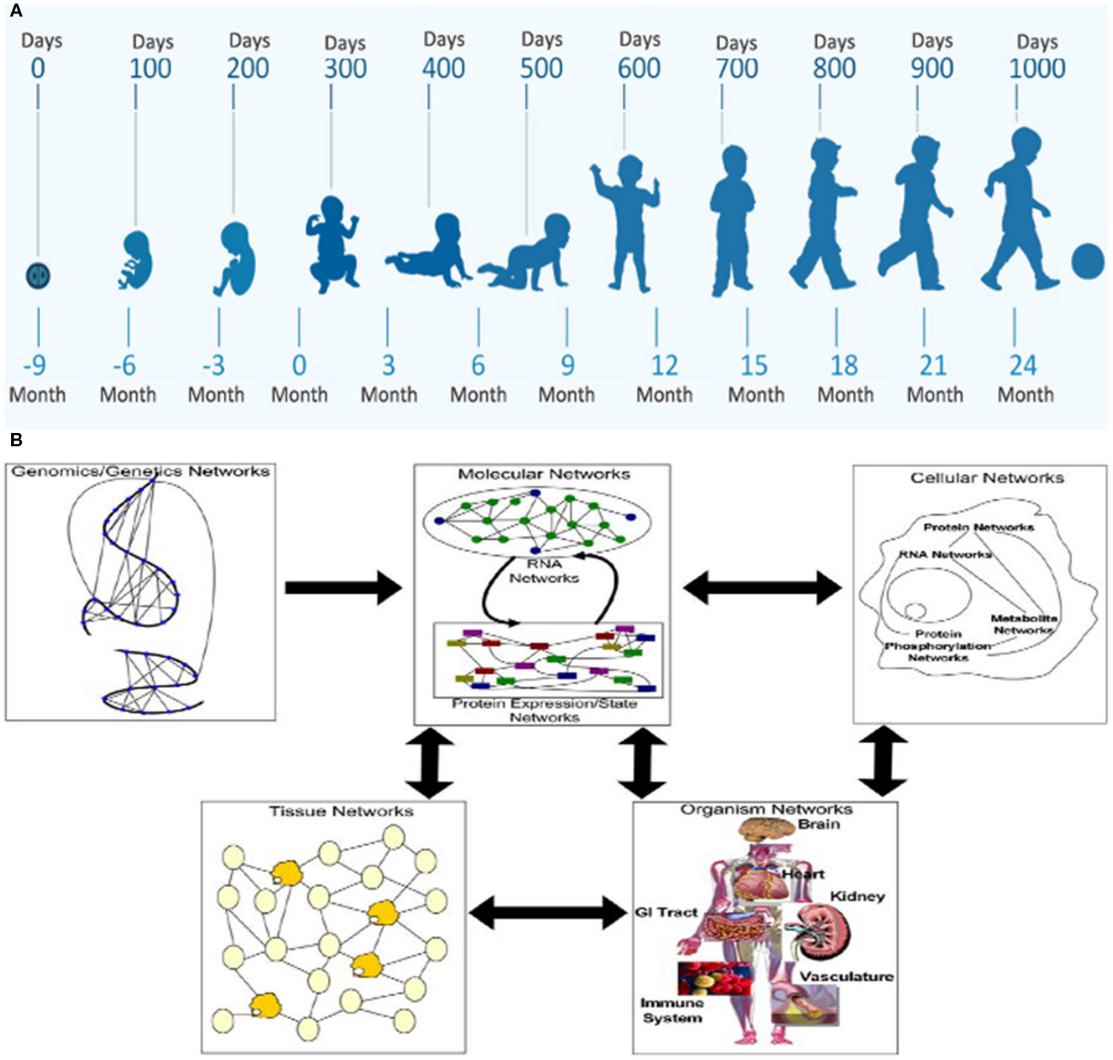
The horizontal/vertical diagnostic process across maturing phenotypes over the first 1,000 days: **(A)** WHO program diagram depicts the horizontal perspective over the first 1,000 days from conception through 2 years of age. **(B)** The vertical diagnostic perspective depicts the flow of information across biological systems through a hierarchy of networks. Each panel highlights a different set of networks at play in a biological system. Genomics networks represent interactions among DNA sequences that may give rise to longer-range as well as more local chromosome structures that modulate gene activity, in addition to inducing synergistic effects on higher-order phenotypes. Genomics networks drive molecular networks composed of RNA, protein, metabolites, and other molecules in the system. Molecular networks are components of cellular networks in which the complex web of interactions among these networks gives rise to the complex phenotypes that define living systems. Tissue networks comprise cellular networks that are clearly influenced by the molecular and genomics networks, and organism networks comprise tissue networks that are clearly defined by the component cellular and molecular networks. Complex phenotypes like disease emerge from this complex web of interacting networks, given genetic and environmental perturbations to the system (26).

MPF triad factors considered by the FNN require horizontal and vertical diagnostic perspectives to optimally anticipate health or disease of the developing brain. Identifying changing phenotypic form and function with maturation over the first 1,000 days comprises the horizontal analytic approach (Figure 1A). From conception, the “patient” is the MPF triad, maturing at successively older gestational ages (GA). Each MPF triad phenotype influences the developing fetal brain, specific to integrated multi-system interactions at a particular gestational age. Systems-biology science applies the vertical analytic perspective at each developmental niche. Gene-to-multi-organ system interactions elucidate interrelated genetic and epigenetic mechanisms that positively or negatively affect the developing nervous system (Figure 1B) (26). This dual diagnostic process guides the FNN to consider a range of brain disorders over the first 1,000 days, with anticipation of neurologic sequelae across the life-span.

Ontogenetic adaptation (OA) is a principle of developmental plasticity, applicable to interdependent systems across species (27). Adaptive responses are system-specific to adverse conditions or diseases during pregnancy that promote survival and optimal maturation. Adaptive nervous system responses to adverse conditions alter brain connectivities appropriate to the developmental stage. While an earlier response during development may be optimal at that age, less effective adaptive responses may later result when new conditions occur at more advanced stages. “Multiple-hits” increase the likelihood that structural and/or functional damage results. Adaptive changes remain permanent during critical/sensitive developmental time-periods, either with positive or negative consequences. Maladaptive neuroplasticity consequently may result in brain disorders, particularly during the first 1,000 days initially expressed by the fetus, neonate, or young child.

Applying OA to this dual diagnostic process enhances the FNN's ability to recognize or anticipate brain disorders during the first 1,000 days. Fetal/neonatal brain disorders are better understood when OA is applied to the evaluation of the maturing MPF triad, starting with the peri-conception time-period. The FNN can then apply this multi-disciplinary knowledge base of the MPF triad to evaluate the GNNS in the neonatal period as well as developmental disorders, epilepsy, behavioral/cognitive disorders, and mental health diseases during childhood, adolescence, and early adulthood. The DSM-5 classification of mental health and neurologic disorders more recently reclassified a wide range of conditions experienced across the life-span that consider regulators of experience-dependent plasticity introduced during the first 1,000 days, with different clinical expressions and potentials for neurologic recovery dependent on the age of presentation. Disease categories that must be anticipated by the FNN are exemplified by autism, schizophrenia, intellectual disabilities, and neurodegenerative diseases, both before and after 2 years of age.

## Fetal/Neonatal Neurology Consultations During the First Half of Pregnancy

First and second-trimester maternal levels of care (28, 29) utilize current testing protocols with benefits and limitations. Abdominal and transvaginal sonographic procedures combined with selected blood studies identify presumably healthy MPF triads based on the absence of major anomalies, aneuploidy, congenital infections, and abnormal fetal growth. FNN referrals more likely are requested when abnormal brain development is suspected with identifiable abnormal results.

Sonographic protocols assess the fetus and placenta for multi-systemic anatomic markers while screening for nuchal transparency at 10–13 weeks GA and organ-system anomalies at 18–22 weeks GA (30). Placental/cord anomalies of implantation, placentation (31) and cord insertion (32) may be detected and monitored. Detection of aneuploidy using non-invasive prenatal testing (NIPS), serologic titers for congenital infections and Pregnancy-Associated Plasma Protein-A Levels (PAPP-A) for FGR (33, 34) are examples of currently useful early pregnancy tests applied to later obstetrical care. The FNN requires an understanding of the uses and limitations of the full range of surveillance tests, while contributing to the diagnosis and intervention for MPF triad disease pathways that adversely affect brain development throughout pregnancy. Educational and research priorities can then be more effectively applied within a FNNP.

**Figure 2:**
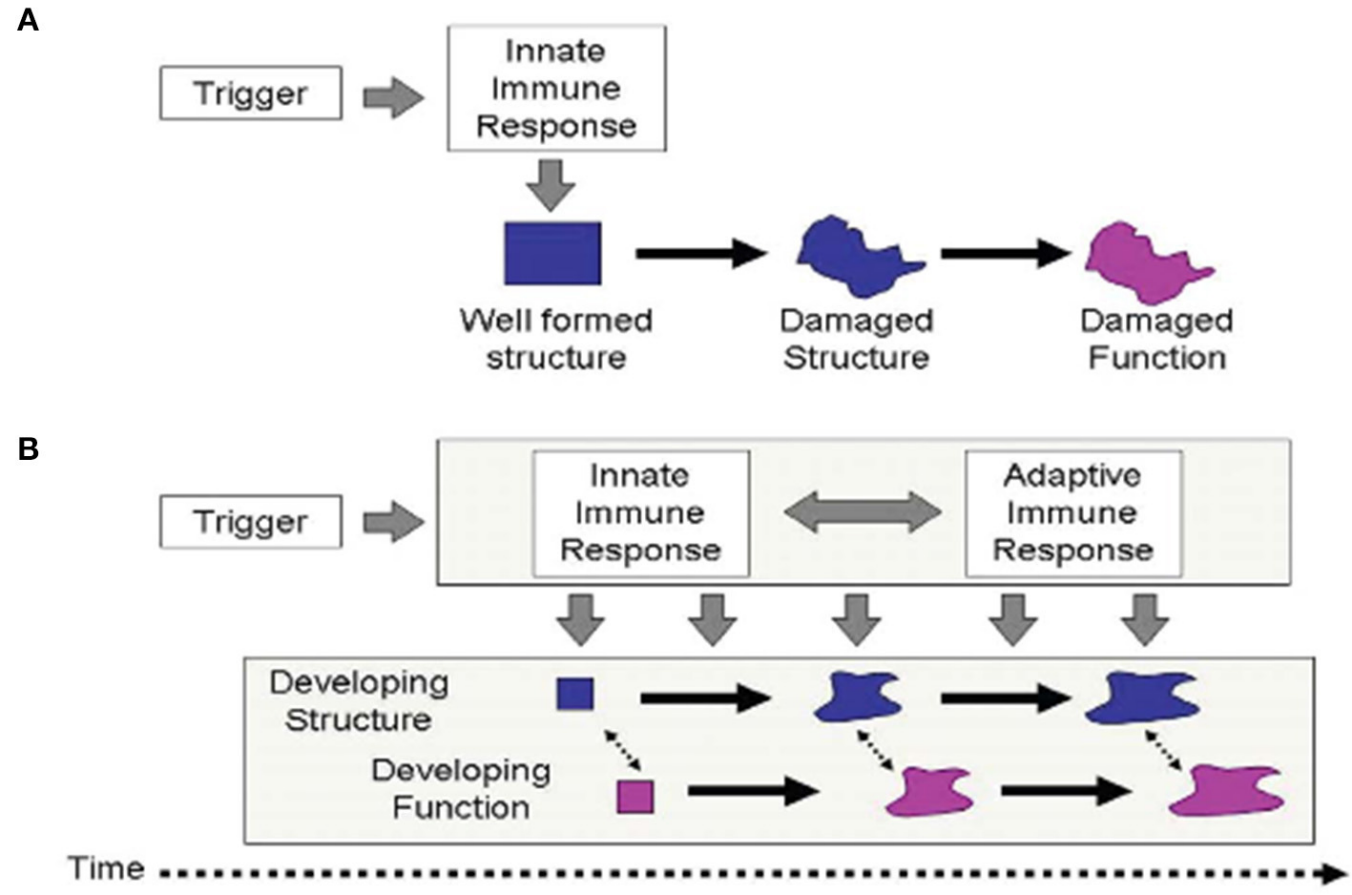
The traditional scenario of perinatal brain injury **(A)** postulates that a single and an abrupt insult damages existing structure, expressed as altered function. This process often occurs from immune intolerance introducing an inflammatory process. Malaeb and Dammann (40) proposes an alternative view **(B)**, which postulates that after an initial trigger has occurred, an ongoing interaction between innate and adaptive immune processes adversely affects the development of structure and function over an extended period of time through infectious or non-infectious inflammatory mechanisms affecting the developing brain.

Immune tolerance between mother and conceptus is required across species to sustain pregnancy and promote healthy development (35). Health balance of the maturing MPF triad's immune system is important for brain development (36). Dysregulated maternal immune tolerance contributes to early fetal loss or abnormal MPF triad maturation. Diverse mechanisms (37) promote imbalance of immune tolerance that affect multiple MPF triad systems, with primary or secondary effects on embryonic and fetal brain structures. Developmental immunological concepts of innate and adaptive immune responses are applicable when considering adverse effects on brain development (38) from inflammatory processes. While a single hit may cause permanent injury, multiple hits alternatively contribute to increasing brain sensitization with cumulative pre-clinical exposures that are later expressed as neurologic sequelae (39) (Figures 2A,B). These processes apply the principles of OA to brain maldevelopment and disease expression from disease processes such as abnormal inflammatory mechanisms. Multiple diseases or environmental conditions such as ascending genitourinary infections, maternal obesity, poor nutrition, toxin/pollulant exposures, and socioeconomic adversities represent infectious and non-infectious inflammatory states that promote immune intolerance across the developing placental interface. Negative effects on brain structure and function beginning at conception over the first 1,000 days may be clinically expressed as developmental disorders such as autism, or beyond 2 years of age as other expressions of developmental disorders such as attention deficit disorder as well as mental health disorders such as schizophrenia.

**Figure 3:**
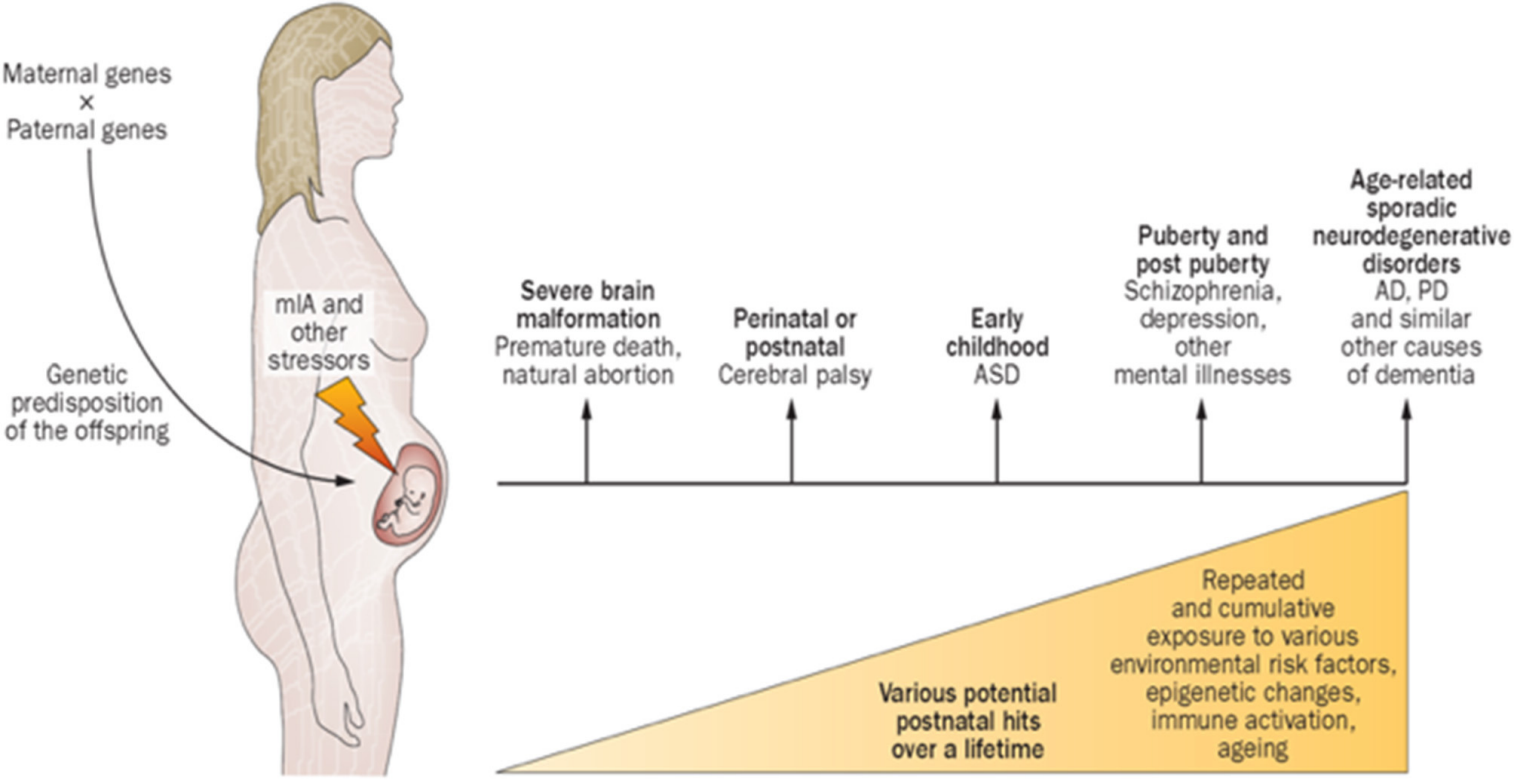
Proposed causal chain of events with mIA in humans, leading to a wide spectrum of neuronal dysfunctions and behavioral phenotypes observable in the juvenile, adult, or aged progeny. AD, Alzheimer disease; ASD, autism spectrum disorder; mIA, maternal immune activation; PD, Parkinson disease (42).

Maternal immune activation (MIA) encompass diseases or conditions involving immune intolerance, implicated with multiple psychiatric and neurological disorders expressed across the life-span (41). Different factors shape the specificity and severity of adverse outcomes. The FNN should anticipate how disorders of immune intolerance impact neuroembryonic and early fetal brain development. Understanding how G × E interactions promote resilience or susceptibility to immune intolerance during the first half of pregnancy provides the FNN with greater diagnostic insight during the remaining “first 1,000 days” and into older ages. Neurologic and psychiatric sequelae such as autism, schizophrenia, intellectual disability, and dementias later expressed during childhood into adulthood will be better anticipated. Future diagnostic/therapeutic advances will foster improved outcomes (Figure 3). Prevalence estimates of MIA are not yet available, but a broad range of pre-conception and early pregnancy infectious and non-infectious inflammatory etiologies cumulatively contribute to this disease process. MIA highlights a major public health priority during preconception and early pregnancy time periods with adverse effects across the lifespan, adversely affecting women during and beyond reproductive years as well as their children (43).

Detection of brain anomalies are limited by the resolution and timing of current testing modalities available to the FNN. Earlier imaging abnormalities utilizing sonography generally require later confirmation by fetal or neonatal MRI studies with greater potential to detect anomalies. Serial studies better document more widespread or subtle nervous system anomalies, particularly in response to multi-systemic diseases of the MPF triad. Major embryonic and early fetal brain or spinal malformations include disorders of neurulation, midline anomalies, segmentation/cleavage defects, and ventriculomegaly (1). These are examples that are more easily detectable by current neuroimaging during the first half of pregnancy. These findings will be integrated into postnatal medical care, anticipating childhood complications requiring treatments such as developmental care interventions, surgical procedures to treat hydrocephalus and brain lesion identified intractable epilepsies. A small minority of fetal patients with myelomeningocele can be currently offered prenatal repair at selected medical centers where fetal neurosurgical expertise is available. A recent prospective intervention study reported decreased need for shunting, reversal of hindbrain herniation, and improved motor function for the prenatal repair group compared with postnatal repair. Pre-existing and more pervasive brain anomalies responsible for more pervasive developmental disorders however cannot be reversed by this fetal repair (44). Molecular genetic diagnoses are (45) presently utilized after birth to improve diagnostic accuracy. Future trimester-specific screening will offer diagnostic approaches during earlier stages of malformation with the opportunity for more effective prenatal therapeutic interventions.

**Figure 4:**
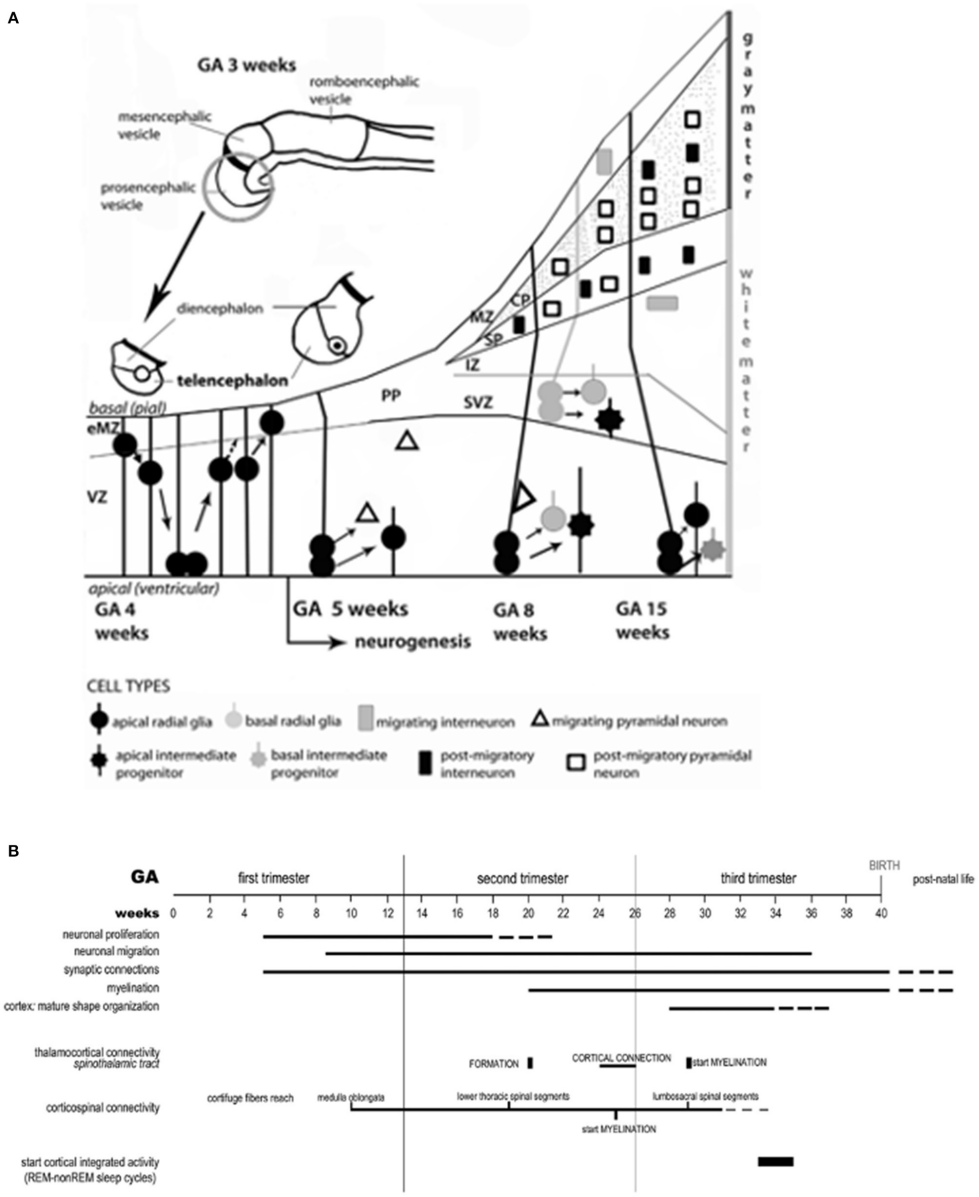
**(A)** The neuro-ontogenic process starts at gestational age (GA) weeks 2–3 with the constitution of the neural tube. At GA week 4, the rostral portion of the neural tube forms the prosencephalic, mesencephalic, and romboencephalic vesicles. The prosencephalic vesicle then forms two vesicles that are destined to become the telencephalon and the diencephalon (thalamus, hypothalamus, and other structures). The schematic diagram represents the development of telencephalon. Initially, the telencephalic primordium is constituted by dividing neuroepithelial cells (often called neural stem cells), characterized by interkinetic nuclear migration, which form the ventricular zone (VZ). There, one type of cells, the radial glial cells, is particularly prominent and distinctive. At GA week 4, they undergo to an early exponential proliferation increasing the number of progenitor/intermediate progenitor cells and the thickness of VZ. Early born neurons are interneurons which move within the marginal zone (MZ) and intermediate zone (IZ). The MZ will eventually form cortical layer I and, for some authors, MZ could be identified also before the cortical plate (CP) formation as early MZ (eMZ). The IZ is a cell-sparse compartment and exists before the appearance of the CP. Radial glial cells and intermediate progenitor cells can be classified in two distinct subpopulations: apical, resident in VZ with bipolar fibers, and basal, which delaminate from VZ with unipolar basal fiber. Apical progenitor cells in the VZ and basal progenitor cells in subventricular zone (SVZ) are considered the main source of pyramidal neurons. Around GA week 5, the neurogenesis begins and the neuronal precursors proliferate rapidly within the VZ. The neurons located in the first recognizable cortical layer, known as the preplate (PP), form the earliest synaptic connections. PP is a transient structure present before the appearance of the CP. Around week 7, the PP cells contribute to the subplate (SP) which remains below the CP after its formation and contains post-migratory pyramidal neurons and interneurons. Moreover, the accumulation of basal progenitor cells creates a distinct new compartment above the VZ, the SVZ. Here, they divide and are considered an additional source of intermediate progenitor cells. By GA week 8, radially migrating neurons from VZ and SVZ initiate the development of the layered CP forming from inside to outside. For example, pyramidal neurons eventually migrate outward along the radial glial cells. **(B)** Timeline of the neurobiological processes in the telencephalon and the sensory/motor connectivity during human ontogeny. The weeks of gestational age (GA) are reported and related to the major events during fetal neural development: neuronal proliferation; neural migration; synaptic connections; myelination; cortex (mature shape organization); thalamocortical connectivity (spinothalamic tract); cortico-spinal connectivity; functionally expressed as cortical integrated activity (REM-non-REM sleep cycles).

Whether isolated or part of syndromic diseases, brain anomalies represent complex MPF triad G × E interactions that profoundly impair transient brain compartments containing neuronal/glial progenitor cell lineages. Figure 4A illustrates the early proliferation, differentiation and migration of multi-potential precursor populations during the first half of pregnancy that originate within transient compartments (46) such as the subventricular region, marginal zone, and ganglionic eminence. Abnormal neuroaxis lengthening and forebrain induction may result from these early alterations. Impaired secondary yolk sac function followed by abnormal placentation negatively alter neuroembryonic followed by fetal brain structures during the 8 weeks after conception. Altered signaling processes such as Wnt pathways (47) promote aberrant abnormal patterning within cell lineages, adversely affecting later axon guidance, synapse formation and neuronal connectivities during the second half of pregnancy (Figure 4B). A growing list of proteins and pathway aberrations are being identified that alter cell positioning, neurotransmitter expression and neuroplasticity responses (47–49) throughout the first half of pregnancy. For example, altered microglial progenitor cells that originate from the neuroectoderm or yolk sac populate embryonic subventricular and marginal zones as well as ganglionic eminence structures. These alterations contribute to abnormal interneurons with suboptimal connectivities (50) during the second half of pregnancy.

Interneurons constitute an important component of brain circuitry, comprising 25–30% of cortical populations in primates (51). Imbalance of excitatory/inhibitory properties by malfunctioning interneurons (i.e., interneuronopathies) will affect mature brain function based on altered early transcriptomics (52), later expressed as a range of neurodevelopmental disorders including intellectual disability, autism, and epilepsy (53). Altered brain development affecting multiple neuronal, glial cell, and angiogenic precursors during the first half of pregnancy consequently will also have profound effects on brain health across the life-span expressed as dementias, neurodegenerative and cerebrovascular diseases.

## Fetal/Neonatal Neurology Consultations During the Second Half of Pregnancy

During the second half of pregnancy, abnormal migration, dendritic arborization, synaptogenesis, and myelination within maturing structures such as the subplate and cortical plate contribute to earlier brain dysgenesis (54) that originated during the first half of pregnancy (Figure 4B). Hypoxic–ischemic, inflammatory, and hemostatic disease pathways in MPF triads during the second half of pregnancy result in destructive brain lesions, often superimposed on earlier dysgenesis (55).

FNN consultations are commonly requested after referrals for high-risk maternal care. Interdisciplinary MFM conferences provide opportunities to include the pediatric neurologist in discussions across specialties that enhance the diagnostic and prognostic perspectives of adverse effects on the developing nervous system. Primary fetal neurologic disorders and secondary systemic conditions of the MPF triad comprise a comprehensive list of diagnostic considerations for the FNN (19). Adverse effects on fetal brain development result from multi-systemic MPF triad disorders. Hypertensive (56), metabolic (57), autoimmune disorders (39), and genitourinary infections (42) exemplify maternal diseases that contribute to experience-dependent maladaptive neuroplasticity that are later expressed as neurologic disorders. Transgenerational (58) and pre-pregnancy maternal health status (23), previous pregnancy loss (59), infertility (60), multiple-gestation pregnancies (61), and toxic maternal stresses (62) expand this list of risks for neurologic sequelae.

Serial fetal surveillance tests monitor structural and functional changes that may reflect worsening fetal brain disorders (63). Fetal growth restriction (64), altered amniotic fluid volumes (65), hydrops fetalis (66), and systemic non-cardiac anomalies (67) exemplify trimester-specific biomarkers associated with potential fetal brain injury. Disease pathways include collectively ischemic placental syndromes (IPS) (68), often superimposed on earlier placental inflammatory disorders resulting from MIA (41). Reduced placental size and/or dysfunction of the developing chorionic villous system compromise fetal nutrient/waste removal and deprive multiple neuroactive substances required for healthy fetal brain development (69). These prenatal considerations need to be considered by the FNN when evaluating the GNNS and early childhood neurologic disorders.

Trimester-specific G × E interactions involving one or multiple systems adversely alter brain development. Congenital heart disease (CHD) is the most common fetal anomaly, serving as an illustrative example. CHD is often associated with fetal brain disorders through multiple mechanisms (70). Genetic pathways result in cardiac and brain malformations after conception, represented by syndromic and non-syndromic disorders. Standardization of clinical care, education, and research have been developed to consider these genetic influences (71). Associations with adverse neurodevelopment require aggressive use of cardiac/brain gene panels to identify survivors whose sequelae began with early signaling pathway defects during the neuroembryonic period (72). These CHD genetic disorders adversely influence later medical/surgical interventions with cumulative brain injuries during the first 1,000 days. Continuity of risk include acquired circulatory-related complications to the fetal brain during the second half of pregnancy that alter childhood and adulthood neurologic function, affecting quality of life, and life expectancy (73).

Tuberous sclerosis complex (TSC) illustrates the challenges and opportunities for the FNN who applies the dual diagnostic process regarding CHD and brain development (74) to one of multiple complicated genetic syndromes considered by the pediatric neurologist. Fetal intracardiac masses such as myxoma and fibroma are often the initial findings that suggest TSC. While cardiac masses often reduce in size or resolve, neurologic phenotypes are later expressed as brain malformations, epileptic encephalopathies, and developmental disorders over the first 1,000 days, even prior to neurocutaneous lesions. Anticipation of TSC by the FNN during early pregnancy will enhance anticipatory guidance for the family regarding sequelae associated with TSC (75) for their child and identify risks to planned offspring in subsequent pregnancies. Future fetal neurotherapeutic interventions may offer metabolic/genetic treatments that mitigate or avoid sequelae ranging from cardiac disease, epilepsy, and autism (76, 77).

Placental/cord maldevelopment are often associated with both genetic and acquired diseases associated with CHD (78). Circulatory-related perfusion brain injuries result from placental vascular disease superimposed on blood flow abnormalities from CHD. Genetically-based placental mechanisms also adversely affect brain development from other MPF triad disorders besides CHD (79). Placental/cord lesions are associated with multi-systemic genetic syndromes or specific organ anomalies disorders such as skeletal dysplasia (80) and gastroschisis (81) with effects on brain development. The FNN must consider shared parental and fetal genetic expressions by the placenta that contributes to brain disorders. Imprinting defects, mosaicism, and epigenetic G × E interactions represent diverse genetic pathways that impair placental development with adverse effects on the brain (82). Postnatal pathological analysis may be the first opportunity to correlate histologically-identified placental lesions such as vasculopathies, dysmaturation, villitis of unknown etiology, and cord anomalies with genetic and acquired MPF triad diseases (83). Future placental genetic/omics analyses and placental imaging modalities (84, 85) will identify MPF triads expressing the great obstetrical syndromes (GOS). These prenatal diagnostic tools will later be compared with placental/cord pathological assessments to help design future neurotherapeutic interventions to reduce neurologic sequelae, presenting either as the GOS or GNNS.

## The Great Obstetrical Syndromes

**Figure 5:**
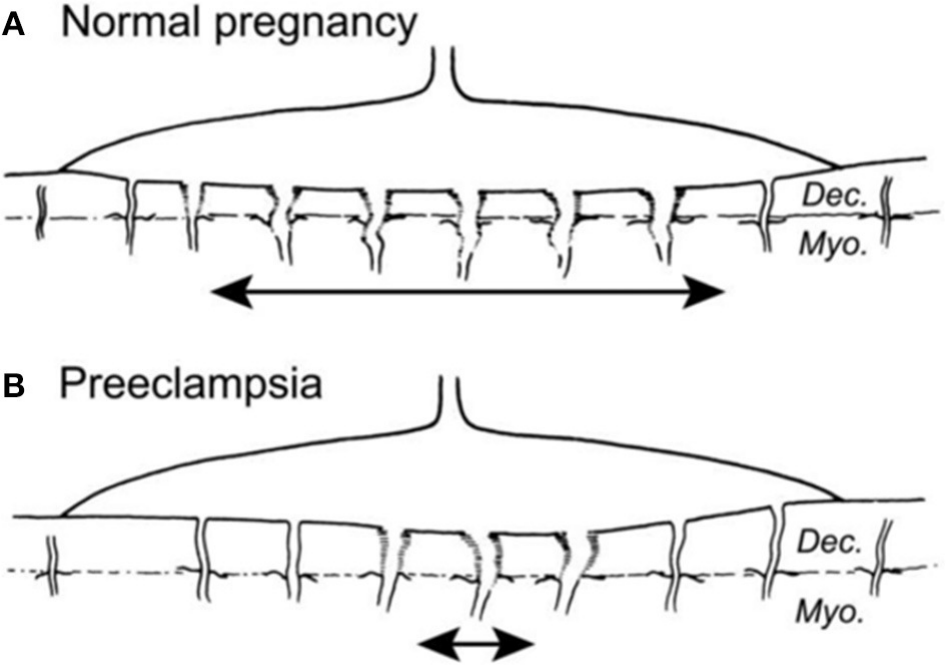
**(A)** Normal placental bed with full transformation of the myometrial spiral arteries except at the periphery of the placental bed **(B)**. Defective deep placentation is characterized by non-transformation of the myometrial spiral arteries reducing the central area with deep placentation (87).

The GOS (86) may adversely affect MPF triad health including fetal brain development. Diagnostic considerations regarding trimester-specific MPF phenotypes associated with the GOS are guided by systems-biology perspectives regarding the developing placental vascular bed. Adverse outcomes include preeclampsia, fetal growth restriction (FGR), prematurity, fetal demise, abruptio placenta, and morbidly adherent placenta. Common disease pathways alter the developing placental vasculature, mediated by G × E interactions of leucocytes and macrophages beginning with trophoblastic development (87, 88) during implantation. GOS are represented by the IPS (68) resulting in shallow and incomplete remodeling of spiral arteries (Figures 5A,B). Chronic asphyxial conditions from the GOS contribute to brain injuries later in the third trimester, during labor and delivery, as the GNNS and during childhood following postnatal illnesses that later further injure brain.

Estimates of GOS suggest involvement of more than 15% of pregnancies, with short- and long-term adverse MPF triad outcomes. GOS represent (1) multiple etiologies; (2) trimester-specific MPF triad factors; (3) maladaptive developmental plasticity, and (4) complex G × E interactions (86).

Biofluids from the MPF triad and neonate contain metabolomic biomarkers that can be useful to diagnose and monitor antepartum, peripartum, and neonatal diseases associated with the GOS. Endogenous metabolites that represent biological systems can assess pregnancy complications associated with GOS (89). Soluble adhesion molecules involved with abnormal inflammatory pathways (90) represent potential diagnostic biomarkers to be applied to neuroprotective interventions during the first 1,000 days. Specific inflammatory mediators such as altered IL-6 allelic expression has been associated with cerebral palsy (91) and exemplify one potential biomarker to treat developmental disorders. Inflammatory mediators such as IL-6 identify MPF triads who are more vulnerable to the multi-systemic sequelae associated with fetal inflammatory response syndrome (FIRS), which commonly mimics hypoxic–ischemic encephalopathy (HIE) (92).

## Peripartum Fetal/Neonatal Neurology Considerations

### Uses and Limitations of Fetal Surveillance Testing

Adverse antepartum conditions impact peripartum events, generally identified as the time-period close to and including labor and delivery (93). Multiple etiologies contribute to fetal distress as detected by biophysical scales, Doppler flow indices, and fetal heart rate patterns, noted during maternal care in the clinic or hospital setting. Obstetrical strategies may be adjusted based on worsening trends or sudden events from these test results. However, fetal distress is often a clinical biomarker representing an adaptive response (94) that signifies fetal brain and selective organ protection. Current testing tools are poor predictors of either past or contemporaneous fetal brain injury. Abnormalities may also reflect physiologic changes that are associated with brain injury that already occurred. Falsely normal results alternatively may be recorded despite pre-existing brain anomalies or injury. While fetal brain injury may be associated with abnormalities closer to the time of a test, the practitioner is unable to distinguish this subset from the majority of MPF triads who either remain protected or who have already been injured.

Physiologic changes during the peripartum period require adaptations for successful fetal to neonatal transition. With entry into the pelvic inlet and descent through the vaginal tract, fetal adaptations are activated that primarily protect multi-organ functions including the brain. All eutherian species activate physiologic reflexes to protect vital organs through delivery with serial uterine contractions. Human fetuses have preserved these protective functions to accommodate larger skulls during descent, crowning, and delivery. These evolutionary adaptations have been maintained across species for survival and health (95), usually requiring minimally assisted delivery procedures for the vast majority of livebirths who escape brain injury.

Electronic fetal heart rate patterns (eFHR) during labor document rhythmic changes during uterine contractions that activate baroreflex and peripheral chemoreflexes. Patterns such as tachysystole or bradycardia may represent transient fetal hypoxic intervals, principally during uterine contractions. Hypoxia primarily activates protective responses and is not predictably associated with contemporaneous brain injury. The peripheral chemoreflex more than the baroreflex (96) effectively protects vital organs for the vast majority of the fetal population (94). Glucose is anaerobically metabolized to lactate as the principal fuel source rather than conversion to CO_2_ and water through the complete aerobic process utilizing oxidative phosphorylation. This reflex has been preserved from lower mammalian species to principally protect brain, heart, and adrenal gland. Progressive worsening of pattern categories cannot reliably differentiate fetal distress with or without brain injury to justify universal recommendations for changes in obstetrical management (97) that will improve outcome. Loss of this protective reflex is often associated with pre-existing MPF triad diseases because of chronic placental pathological lesions (98), as represented by the GOS. In the absence of antepartum vulnerabilities from MPF triad diseases, these protective reflexes primarily provide protection from HI brain injury. Situations leading to injury depend on the pathophysiological form and duration of HI during the peripartum period in the context of pre-existing MPF triad vulnerabilities.

Three forms of fetal hypoxia have been applied to experimental design models to assess when and how brain injury may result. The most common and least injurious form is hypoxic-hypoxia. Based on laboratory findings, this hypoxic state may even precondition specific fetal brain regions and provide complete or partial neuroprotection (99). Two other hypoxic states more often result in brain injury, primarily because of the ischemic component (100). These two states have been studied across mammalian species to demonstrate an evolutionary-stable duration of protection from rodent to primate models before brain injury commences. The experimental methodology in the laboratory can be rigorously controlled by reducing or depriving oxygen and blood flow such as using carotid ligature techniques (i.e., Rice-Vannucci method). A 30-min window of continuous HI is required before specific patterns of cortical and subcortical white and gray matter brain injuries begin. This second HI state has been described as the partial prolonged asphyxial pattern, with injury to cortical and basal ganglia structures. The third ischemic state, termed acute profound asphyxia, requires only 8–12 min duration before permanent injury begins. This third form is more severe, injuring subcortical gray and white matter regions including the brainstem and spinal cord, often resulting in death. Brain region-specific genetic resilience or vulnerability is an additional challenge to predict the timing and etiologies associated with either form of HI brain injury. This genetic heterogeneity will require future patient-centered diagnostic tools for identification of more effective therapeutic interventions (99).

In the clinical setting, these three hypoxic states are not mutually exclusive with overlapping occurrence, and may be expressed postnatally as hypoxic–ischemic encephalopathy (HIE). Specific MPF triad factors contribute to the individual patient's pattern of injury after variable durations and severities of peripartum asphyxial stress. The animal models described above define duration and patterns of injury for strictly HI in controlled laboratory settings.

More complex experimental disease models, representing alternate etiologies may either accompany or mimic HIE. One common disease mechanism is expressed after birth as a neonatal encephalopathy (NE) resulting from the fetal inflammatory response. This brain disorder is termed fetal inflammatory response syndrome (FIRS), to be distinguished from HIE. This is a non-modifiable antepartum or intrapartum condition despite antibiotic treatment, given the inflammatory-mediator responses. This disease process often results from an ascending vaginal tract infection, culminating in inflammation of the placental and/or umbilical membranes. While maternal fever, uterine tenderness, foul-smelling amniotic fluid, and leukocytosis with a predominance of immature neutrophils may present as clinical signs, no intrapartum or postpartum signs of infection may present. Histological confirmation of chorioamnionitis and/or funisitis by placental/cord analysis may be the clinician's first opportunity to consider this pathophysiologic mechanism. Brain injury resulting in FIRS may have occurred prior to as well as during labor and delivery, expressed non-specifically as NE (92).

Antibiotic administration does not effectively impede or reverse the FIRS-related injuries given the complex pathophysiologic mechanisms associated with this condition involving inflammatory mediators. Dual mechanisms that are asphyxial and inflammatory-mediator (101) contribute to brain injury. Inflammation produces HI given vasoconstriction, reducing oxygen and glucose delivery to fetal brain within placental/cord vasculature as well as across the blood–brain/blood–CSF barriers. The inflammatory component of FIRS results from disruption of the maternal–fetal immunologic balance. Release of excessive types and amounts of inflammatory mediators such as cytokines occur. These substances worsen the asphyxial injury within the placental/cord vasculature as well as the neurovascular unit of the blood–brain barriers. These substances also breach the neuronal membrane and injure or destroy cytosolic organelles such as the mitochondria as well as alter or destroy genetic material within the nucleus (102). Excitotoxicity from only HI also releases harmful inflammatory mediators which worsen FIR-induced injury. Both sources of excitotoxicity activate microglia which adversely affect neuronal populations in different brain compartments (103). Combined experimental models of FIRS and asphyxia demonstrate more severe brain injury than when occurring separately (104). These models do not follow the classical HI models of brain injury associated with acute profound or partial prolonged asphyxia (100) as expressed by specific neuropathological or neuroimaging findings. Altered obstetrical management in response to eFHR does not predict or avoid brain injury later expressed as FIRS. Expression of FIRS for preterm neonates is more prevalent given a greater risk for chorioamnionitis/funisitis, and also associated with a greater risk for brain injury.

Other non-modifiable etiologies may be expressed later as NE and also mimic intrapartum HIE (105). Genetic loss or gain of function predisposes the MPF triad to fetal brain anomalies during early pregnancy with vulnerability to injuries after later adverse antepartum or peripartum events. Universal newborn screening includes a panel of specific metabolic/genetic disorders that may represent HIE mimics, such as biotin deficiency and sulfur-containing aminoacidopathies. Nutritional or therapeutic treatments prevent or lessen sequelae for selected disorders such as PKU and thyroid disease. However, present testing lack specificity, sensitivity, and timely therapeutic applications. Childhood presentations with cerebral palsy, autism and early-onset epileptic encephalopathies before 2 years of age may combine genetic diseases that contribute to earlier brain anomalies with later vulnerabilities, contributing to destructive injuries later during pregnancy (106–108). Future trimester-specific and postnatal genetic screening using transcriptomic/proteomic biomarkers will identify the susceptible fetus or neonate who might benefit from earlier and more effective preventive, rescue, or reparative treatment interventions.

### Sentinel Events From Maternal/Placental/Fetal Triad Disorders

Sentinel events represent sudden fetal stress associated with hypoxia/asphyxia (HI), often in proximity to labor and delivery. eFHR is unfortunately a poor proxy to reliably differentiate distress with or without brain injury even after a sentinel event, given the protective adaptive responses provided by the peripheral chemoreflex. Intrapartum sentinel events for MPF triads in specific situations can be associated with fetal brain injury (109), despite neuroprotective rescue with therapeutic hypothermia (THT) (110). Multiple antepartum and/or intrapartum MPF triad factors elucidate the complex timing and mechanisms leading to brain injury (15) that indicate why these events are not as yet predictable. While two broad distributions of injury previously described as acute profound or partial prolonged patterns after HI may be documented on later neuroimaging or neuropathology, more complex interactions from multiple etiologies over time may have cumulatively contributed to these brain lesions beyond current testing to detect, classify, or predict outcome.

Abruptio placenta and morbidly adherent placenta (31) represent the first of three sentinel categories. First-trimester placentation disorders associated with later IPS may later only present as intrapartum sentinel events, but are often associated with the GOS (87). Sudden placental membrane separation with hemorrhage during labor and delivery may be the first expression of shallow and incomplete spiral artery remodeling earlier during pregnancy after chronically acquired or genetic etiologies, presenting as abruptio placenta (111–113) or morbidly adherent placenta (114, 115).

A second sentinel category includes umbilical cord lesions. Thrombosis (116, 117) or rupture (118) may be associated with anomalies of cord length (119, 120), insertion (118), knots (121) and coiling (122). Prolapse has been associated with multiparas pregnancies, abnormal presentations, polyhydramnios, and multiple-gestation pregnancies (123). Marginal (i.e., <3 cm from the placental margin) and velamentous cord insertions are first-trimester anomalies, and may only present as acute separation from the placental bed with exsanguination during delivery. These lesions also are associated with IPS despite acute presentations.

Primary and secondary uterine lesions represent a third sentinel category, associated with risk factors based on anatomical defects (124, 125). Myometrial anomalies with or without prior pregnancies (126) as well as chronic lesions from previous uterine trauma (125) predispose to acute intrapartum rupture with hemorrhage during labor and delivery. Leiomyoma, myometrial defects, large uterine lakes (127), and uterine scarring exemplify long-standing uterine lesions that later present as sentinel events with an acute rupture. Pathological examination of the uterus is essential to assign the most accurate etiology and timing for these events based on histopathology.

Whether fetal distress presents as a progressive worsening situation or a sentinel event, adjustments in obstetrical management, neonatal resuscitation, and the application of neuroprotective rescue procedures such as THT are offered. Erythropoietin has been more recently investigated as a supplementary option to THT (128). Antepartum MPF triad risks or diseases are often not fully appreciated when these rescue interventions are initiated. Diagnostic assessments during neonatal neurocritical care may only later identify disease processes that represent antepartum and/or intrapartum diseases, expressed as the GNNS.

## The Great Neonatal Neurological Syndromes

GNNS are complex phenotypes that share features with the GOS, based on trimester-specific G × E interactions affecting the MPF triad. Differences from GOS include (1) the rapid presentation during peripartum and neonatal time-periods, (2) cumulative distal and proximal factors affecting the MPF triad and neonate over the entire pregnancy or after delivery, and (3) phenotypic expressions of brain disorders are often not detectable before birth. Four broad categories of the GNNS are neonatal encephalopathy (NE), seizures (NS) or stroke (NSK), and encephalopathy associated with conditions of prematurity (EP).

Antepartum and peripartum diseases may negatively impact outcome despite adjustments in obstetrical management closer to delivery, as well as resuscitative interventions and neurocritical care for newborns expressing the GNNS (1, 93). Horizontal and vertical diagnostic perspectives guide the FNN to re-evaluate each encephalopathic neonate as additional clinical and testing information emerges over hospitalization. Acute infections, metabolic-toxic, and traumatic etiologies may best explain peripartum or neonatal brain injuries expressed as the GNNS. However, trimester-specific MPF triad G × E interactions worsen or mitigate peripartum and neonatal disease expressions. Childhood neurologic disorders will later present despite neonatal neurocritical care interventions or in the absence of the GNNS. Each phenotype will be separately addressed, although multiple categories are often expressed by the same neonate.

### Neonatal Encephalopathy

**Figure 6:**
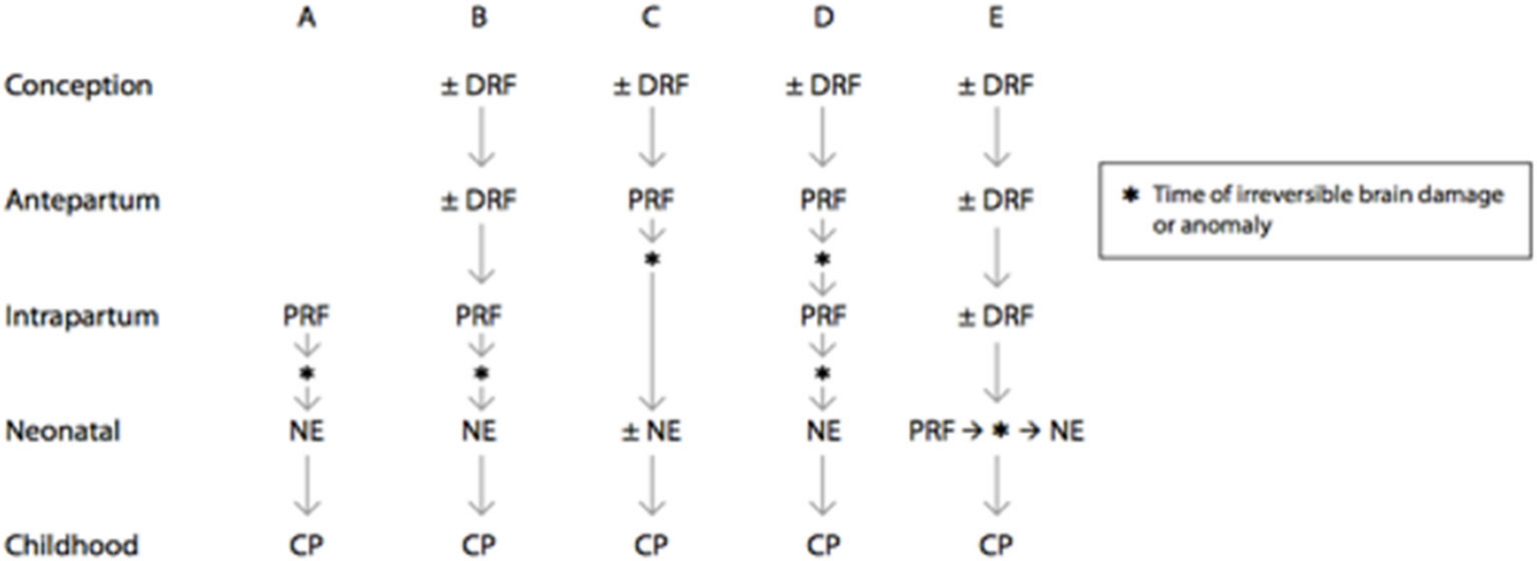
Prenatal and perinatal causal pathways to cerebral palsy in term infants. Distal risk factors exert a pathogenic effect on fetal brain development starting at a time that is remote from the onset of irreversible brain injury. Examples include genetic abnormalities, environmental, and sociodemographic factors, as well as specific placental abnormalities. Proximal risk factors exert pathogenic effects on fetal brain development at a time that closely predates or coincides with the onset of irreversible brain injury. Examples include abruptio placentae, chorioamnionitis, and twin–twin transfusion. There are multiple potential causal pathways that lead to cerebral palsy in term infants, and the signs and symptoms of neonatal encephalopathy may range from mild to severe, depending on the nature and timing of the brain injury. **(A)** Intrapartum brain injury that is due to a proximal risk factor may lead to neonatal encephalopathy and subsequent cerebral palsy. **(B)** Intrapartum brain injury may be the result of both distal and proximal risk factors that predispose the fetus to brain injury and cerebral palsy. **(C)** Brain injury or anomaly may occur in the antepartum period as a result of distal and proximal risk factors. When brain injury or anomaly occurs at a time that is remote from the delivery process, neonatal encephalopathy may or may not be seen after birth. **(D)** Brain injury may occur at multiple points during gestation. **(E)** Proximal risk factor and brain injury may occur in the neonatal period following predisposing distal risk factors. DRF, distal risk factor; PRF, proximal risk factor [(15), updated 2019].

NE occurs in 1–3/1,000 term and near-term livebirths. Multidisciplinary consensus reports reaffirm and update scientific studies that associate NE with neurologic sequelae (15). Five clinical pathways include distal and proximal trimester-specific MPF triad factors that cumulatively result in NE (15) (Figure 6). Accurate diagnoses of NE have patient-centered, health policy and medicolegal ramifications (129). Half of those with NE are associated with HI, with more than 70% occurring during antepartum or peripartum time-periods. Less than 10% of brain injuries occur closer to delivery but may still be associated with trimester-specific MPF triad etiologies that increase risk.

Neonatal depression after delivery may evolve into NE. Resuscitative interventions followed by evolving clinical repertoire over time include a range of signs and symptoms required to define NE. Sustained altered arousal and muscle tone as well as seizure expression are associated with multiple NE phenotypes based on timing and etiology. Serial clinical neurodevelopmental assessments (130), multi-systemic evaluations (131), serial EEG/neuroimaging and placental/cord examinations cumulatively reveal more precise estimations of timing, etiologies, and prognosis for HIE or HIE mimics (1). However, the most accurate assessment may not be apparent given trimester-specific MPF triad factors that escape current diagnostic testing. Initial clinical scores (i.e., Apgar and Sarnat scores) assign NE severity and eligibility for neuroprotective rescue without complete understanding of the multiple expressions of NE (132, 133) based on trimester-specific MPF triad disorders.

Neonates with minimal resuscitative needs contrasted with the suboptimal responses by stillborns and apparently stillborn survivors represent two ends of the NE spectrum. The fetus who recovers from non-lethal antepartum brain injury may subsequently negotiate labor and delivery with less significant fetal distress and neonatal depression and appear less severely encephalopathic. These children require more limited resuscitation and escape injuries closer to delivery. Given their improving clinical status, this group does not strictly qualify for THT based on the randomized controlled trials (RCT) (134) that treat presumed HIE. Proposed RCT protocols for this mild NE population are now being studied, some of whom might benefit from neuroprotection (135, 136). More effective diagnostic tools, however, are needed that can sufficiently distinguish those vulnerable newborns with mild NE who would benefit from THT because of the onset of HI within the recommended 4- to 6-h window to maximize treatment efficacy. THT would alternatively not be beneficial for two other “mild NE” groups who either successfully “auto-resuscitate” without brain injury or those who already suffered earlier injury despite appearing mildly encephalopathic. Peripartum biomarkers have been proposed that might differentiate acute from chronic oxidative stress (137) or epigenetic changes (138), and more accurately identify those with mild HIE who would benefit from THT.

Stillborns (139), a percentage of apparently stillborn neonates (140), and neonatal deaths (141) constitute the severe end of the NE spectrum. Chronic, subacute, and acute structural brain injuries have been documented in all these groups based on neuropathological (142), neuroimaging (143), and placental examinations (144, 145). Risk factors for fetal demise vary across three trimesters (146), and represent one of the GOS associated with diseases of the placental vascular bed. Failure to respond to neonatal resuscitation may reflect antepartum as well as peripartum lethal brain lesions, despite aggressive resuscitative interventions (147, 148). Factors influencing these adverse outcomes include obstacles to obstetrical and resuscitative medical services either in developed or developing countries. Prematurity, infections and brain/systemic anomalies are more prevalent conditions in resource-poor countries.

Serial clinical examinations help distinguish intrapartum HIE from mimics. However, diagnostic challenges exist as a result of complicated evolving postnatal illnesses or responses to treatment interventions. Neonates may appear more encephalopathic such as with worsening cardiorespiratory disease or infection. Treatment interventions such as phenobarbital and THT may be required but unfortunately alter tone/arousal and also impede the diagnostic process. Serial expressions of arousal, muscle tone, and seizure onset/severity may alternatively suggest an earlier disease process. Rapidly improving Apgar scores followed by normal neurological examinations or mild clinical severity scores generally do not support clinical repertoire associated with late intrapartum HI close to delivery.

Hypotonia and early seizures are generally expressed after significant intrapartum HI. Acute early hypertonia alternatively may reflect the mild “Sarnat 1” clinical severity score, infection, intracranial hemorrhage, abstinence signs or pain. Hypertonia following intrapartum HI is generally anticipated to be expressed after 5–7 days of life. Earlier expression of hypertonia alternatively reflects chronic acquired (149, 150) or genetic disorders (151), once acute etiologies such as infection, drug withdrawal and intracranial hemorrhage are eliminated as etiologies. In the absence of these acute scenarios, hypertonia can be associated with the over-expression of subcorticospinal pathways after significant chronic or subacute cortical injury or maldevelopment. A recent rabbit pup model described neuroimaging (152) and neuropathological examination (153, 154) findings that reaffirm earlier primate studies (155, 156). With or without early hypertonia, early NS onset with or without NE is another clinical sign that may represent antepartum etiologies (157, 158).

Multiorgan clinical assessments compared with neurological examinations help assign timing and etiology to the disease process. Symmetric and asymmetric patterns of FGR (159), microcephaly (160, 161), facial/body stigmata (1) are important examples to consider. Optic nerve hypoplasia, retinal coloboma, and cutis aplasia are examples of specific first-trimester optic vesicle maldevelopment and neuroectodermal markers of brain dysgenesis. Dysmorphic facial features, arachnodactyly, and body size abnormalities more generally exemplify trimester-specific stigmata associated with syndromic or non-syndromic brain maldevelopment (1). Serial examinations throughout the neonatal hospitalization after removal of supportive medical equipment offer greater opportunities to document subtler and less obvious examination abnormalities.

Multi-organ testing may reveal patterns of dysfunction or injury that support NE timing and etiologies (131). Specific laboratory abnormalities suggest intrapartum and/or antepartum HI, infectious/non-infectious inflammatory states or hemostatic diseases. Specific organ involvement immediately following resuscitation raise suspicions of antepartum MPF triad diseases associated with brain injuries (1): (1) normal or mild renal/hepatic dysfunction (131), (2) elevated nucleated red blood cell counts (162), (3) early cardiac chamber or septal hypertrophy (163), (4) persistent pulmonary hypertension expression based on pulmonary vascular remodeling or pulmonary hypoplasia (164), (5) immune or non-immune fetal hydrops fetalis (66), and (6) genetic disorders definable in the newborn period, such as coagulopathies (165) or channelopathies (166).

Serial bedside EEG studies enhance physical examinations as early as during triage following resuscitation (167). Recording and interpretation require a skilled neurophysiologic team in cooperation with the neonatal intensive care staff. High-quality studies will be hampered by the sick neonate's need for supportive medical equipment, isolette barriers, medical interventions, and medications. EEG provides important functional assessments that complement structural correlates using neuroimaging. While EEG/sleep patterns are sensitive to the severity of brain disorders, interpretations are not disease-specific. Timing of a brain disorder expressed on the EEG requires the clinical context with historical perspectives, serial physical examinations and an integrative profile applying all relevant study results (168). Specific EEG findings may have neuropathological correlates (169) suggesting acute or chronic brain lesions.

Conventional EEG studies with a full complement of electrographic and polysomnographic signals remain the gold standard. Amplitude-integrated EEG (aEEG) provides important preliminary information regarding the severity of the NE as well as NS diagnosis and burden. Technological advances over the last 50 years have resulted in sophisticated devices applied by trained health personnel that integrate protocols into neonatal neurointensive care (170). While the intubated neonate with NE requiring time-intensive medical interventions better tolerates one or two aEEG channels, strategically timed conventional EEG studies are invaluable to verify epileptic from non-epileptic clinical signs, hemispheric/regional asymmetries or focal electrographic findings of clinical significance (167). As the neonate emerges from the earlier stages of NE to express sleep state transitions, interpretation of quiet, active, and transitional sleep states by conventional EEG/polygraphic recordings depicts persistence or resolution of more pervasive expressions of NE, including physiologic dysmaturity (171). Preservation of EEG/sleep patterns despite neonatal depression shortly after delivery supports a clinical impression that a significant NE has not recently occurred. Brain dysmaturity alternatively may be expressed acutely or over time that suggest a more chronic fetal brain disorder. Abnormal interpretative findings include persistently immature EEG patterns compared with the corrected GA (172) or altered sleep state percentages (173). Interpretation of polysomnographic signals such as autonomic measures, rapid eye movements and other somatic signs also may not agree with the electrographic expression of sleep state for the assumed GA, termed dyssynergia (167), as an expression of a chronic brain disorder.

Serial neuroimaging interpretation using cranial ultrasound (US) followed by brain magnetic resonance imaging (MRI) help correlate timing of brain injuries, although HIE mimicry such as FIRS or genetic disorders may alternatively be the predominant etiology (174), depicted by the same neuroimaging findings.

US is operator-dependent, and requires close communication between sonographer and radiologist. Use of the US better visualizes white matter echogenicity, cystic changes, and hemorrhage particularly for the preterm neonate. Although convenient as a bedside tool, challenges include proper transducer use, size of the anterior fontanelle, and limitations in resolution and field of brain structures that can be visualized. Serial US images beginning during the first 24 h help approximate timing and evolution of a disease process. The acute phase occurs over the first week of life. Symmetric or near-symmetric white matter congestion (edema) is interpreted as increased periventricular echogenicity 24–48 h after the onset of injury. Mass effect with worsening edema peaks on day 3 post-injury with maximal ventricular compression, effacement, or non-visualization of sulcal–cisternal spaces. The subacute phase occurs during 2–6 weeks, with variable degrees of white matter volume loss, gliosis, and cystic formation, with symmetric or asymmetric ventricular enlargement, widening of sulcal-cisternal spaces and resolution of echogenicity. Periventricular cystic formation will more likely be noted for preterm neonates while subcortical cystic formation is noted for term and near-term newborns. The chronic phase after 6 weeks is marked by continued atrophy and cystic collapse. Cerebral edema, liquefaction necrosis, and atrophy during the early phase support a longer time-course associated with injury from multiple etiologies.

Brain MRI is a more sensitive imaging modality with greater resolution and a larger field of view. For neonates near or at term GA, diffusion-weighted image abnormalities (DWI) appear during the first 24 h after injury and precede T1 and T2 weighted sequence image abnormalities. DWI peak changes occur over 3–5 days with pseudo normalization, by 8–10 days without THT, and 10–14 days with THT. T1 and T2-weighted sequences are more useful for timing of an injury. T1-weighted changes occur as early as 2–3 days as a hyperintense signal. After 6–7 days, T2 signals shorten with a darker signal, followed by T1 hyperintensity during the chronic phase. Early T1 or T2 changes before a week after an injury, laminar necrosis/liquefaction necrosis, Wallerian pathway degeneration, or atrophy before 2 weeks suggest an earlier time course that may include the antepartum time-period.

The principal challenge before obtaining an MRI is the need to transport the neonate to a distant hospital location. Specific transport procedures and non-magnetic supportive devices hamper earlier use until the child can be more easily studied when more clinically stable. Minimizing movements require swaddling with or without sedation. Most neonates will consequently have an MRI performed after completion of THT and following extubation, unless compelling clinical reasons require earlier investigation. An alternative imaging modality is a brain CT scan, offering less detailed images but requiring a shorter time to complete the study, sometimes yielding useful information such as intracranial hemorrhage or major brain malformations.

Placental/cord examinations begin with on-site gross inspection at delivery with more definitive evaluations later during the hospitalization. Observations of hemorrhage, the amount of blood loss, and uterine/cord rupture might be helpful. Gross inspection requires color, shape, trimmed weights, true cord lengths, location of cord insertion, number of nuchal cords, and coiling index. An international classification (175) offered terminology for microscopic descriptions of the placenta and cord referring to findings consistent with maternal and fetal vascular malperfusion, delayed villous maturation, patterns of ascending intrauterine infection, and villitis of unknown etiology. Microscopic sections of the cord require details of vessel number and structure, funisitis, as well as edema, volume, and cellular content of Wharton's jelly. This classification better defines the significance of lesions associated with adverse pregnancy and pediatric outcomes. Diverse acute and chronic lesions are associated with all categories of the GNNS. Gross and microscopic examinations of uterine/adnexal structures are essential to document anomalous and destructive lesions. Family counseling regarding the diagnosis and prognosis of their child with a brain disorder should incorporate placental/cord/uterine evaluations into ongoing discussions. Limits of resolution using standard microscopic/staining examinations must also be anticipated. Novel staining techniques may be employed that identify more specific histopathological processes such as iron staining and viral inclusions. Future prenatal and postnatal transcriptomic/proteomic markers as well as prenatal placental imaging modalities (85) will identify more complicated G × E interactions causing diseases, even without pathological lesions.

### Neonatal Seizures

The incidence of NS has been reported as 1–3/1,000 livebirths for term newborns, and 5–6/1,000 livebirths for preterm neonates. Reported percentages vary based on the populations studied and the reliance on clinical or electrographic criteria (176). NS are considered emergencies requiring prompt treatment to prevent secondary brain injury, particularly with reduced cardiovascular perfusion effects on cerebral blood flow from NE/NS-related dysautonomia. NS more commonly is a complex phenotype with trimester-specific G × E factors contributing to expression (176), with or without intrapartum complications. Time and brain-region specific associations encompass diverse etiologies affecting the MPF triad. A multi-dimensional approach to NS better guides the FNN to relevant diagnostic/therapeutic interventions and outcome prediction (176).

NS more likely occur within 1–3 days after birth, usually in the context of fetal distress associated with NE (158). NS can also present as an isolated clinical sign or as the initial expression of an acute neurologic disorder with metabolic/toxic disturbances, intracranial hemorrhage, stroke, or infection at any time during hospitalization.

Synchronized video electroencephalographic monitoring (VEEG) (177, 178) differentiates “epileptic” from “non-epileptic” seizure expression. VEEG provides the most accurate start and endpoints for cortically generated seizures and guides treatment choices. Clinical signs with electrographic events include subtle, clonic, tonic, or myoclonic presentations. Subtle signs are challenging given minimal or atypical expression including dysautonomic signs (167), underscoring the importance of coincident EEG confirmation. Reliance on EEG after antiepileptic medication (AED) administration is essential, given the persistence of electrographic seizures despite the resolution of clinical seizures (i.e., uncoupling) (179). Synchronous and non-synchronous occurrence of clinical and electrographic seizures during the same recording (i.e., dissociation) suggest propagation from brain regions distant from the recording electrodes (180).

Non-epileptic neonatal movement disorders are principally expressed as tremors, myoclonus, and dyskinesias. These disorders are poorly responsive to conventional antiepileptic medications (AEDs). Specific non-epileptic paroxysmal events may be symptomatic without brain lesions such as tremors, while other clinical expressions reflect abnormal brain circuitries throughout the neuroaxis from remote or recently acquired injuries, with or without identification of genetic disorders (176). Neonatal myoclonic movement disorders exemplify the range of diseases from encephalitis to glycine encephalopathy, expressed with or without electrographic correlates (181). Non-epileptic movement disorders require the same rigorous classification as NS (182), given the diagnostic importance of these clinical signs as expressions of trimester-specific MPF triad disorders with varying prognosis.

Pharmaco-resistance to the commonly-used AEDs phenobarbital and phenytoin is often encountered (183). Poor efficacy continue to be reported over a wide range of newer AEDs (184). More rigorous design of therapeutic trials have been proposed requiring more critical comparisons (185), but G × E interactions and trimester-specific MPF triad disorders influence treatment efficacy. Brain maldevelopment from inherited or *de novo* mutations may contribute to poor AED responses, particularly when superimposed on destructive diseases as pregnancy complications occur. Abnormal placentation beginning during first trimester contributes to the later GOS, expressed postnatally as NS, with or without NE. Genetic disorders associated with NS will be more commonly identified by molecular biomarkers either during prenatal or postnatal life (186).

Comprehensive evaluations include serum and spinal fluid testing for aminoacidopathies, organic acidurias, fatty oxidation defects, lysosomal, and peroxisomal disorders. These are obligatory studies to perform although frequent negative results are encountered given their rare incidence. Treatable and non-treatable forms of neonatal encephalopathies with NS should be promptly considered after AED intractability is observed (187). Malformations may be associated with some disorders but current neonatal neuroimaging modalities may lack the resolution to detect abnormalities. Examples of discernable structural findings include different expressions of cerebral dysgenesis from prominent anomalies of midline or lateralized brain structures to more subtle findings of focal cortical dysplasia (176). Serial imaging over the first 1,000 days may later document dysgenesis not noted during the neonatal time-period. Placental/cord examinations should always be part of the evaluation to differentiate antepartum from peripartum etiologies associated with NS in the context of NE (188).

Epileptic encephalopathies are now better-defined as etiology-specific electroclinical phenotypes, historically identified as Ohtahara and early myoclonic encephalopathy (189). Although once considered rare diseases, these syndromes represent a broader range of genetic diseases that contribute to NS with anomalous and destructive lesions, given G × E interactions. Genetic disorders also contribute to greater vulnerability to acquired disease after HI, infection or hemostatic disorders. Genetic epilepsies are associated with anomalous development as a direct consequence of the genetic mutation from conception, in addition to the harmful effects of ongoing epileptic activity on fetal brain development during pregnancy. Loss or gain of genetic function express fetal and neonatal brain disorders may accompany trimester-specific MPF triad diseases and contribute to acquired brain injuries closer to delivery. A genetic mutation associated with NS and NE may be later associated with children who present with developmental disorders such as autistic spectrum (190) and cerebral palsy (106), as well as epileptic syndromes such as infantile spasms (191).

Although only rarely reported based on women's awareness, sonographic documentation or abnormal eFHT, fetal movements may signify *in utero* seizures (192). Normal state-specific movements and hiccups must not be confused with non-epileptic movement disorders or fetal seizures. Examples of fetal seizures with genetic or acquired MPF triad disorders include pyridoxal 5′ phosphate deficiency, MPF triad diseases causing HI and congenital infections.

Development of next-generation genomic sequencing techniques will lead to therapeutic interventions for diverse fetal and neonatal brain disorders associated with the MPF triad. This testing will be used to evaluate postnatal expression of NS with or without accompanying NE. Channelopathies exemplify one class of genetic disorder. Abnormal function of neuronal membrane-bound receptors promote abnormal transport of sodium, potassium, chloride and calcium ions, associated with childhood epilepsies (193). These disorders may be expressed as NS, illustrated by the chloride transporter.

The chloride transporter is an ubiquitous molecular candidate associated with normal and abnormal brain development during the critical periods over the first 1,000 days. Chloride transporter development maintain excitatory/inhibitory balance for healthy development within brain and spinal cord circuitry (194) across three trimesters. The neurotransmitter gamma-amino-butyric acid (GABA) regulates neural activity by modulating neural excitability through chloride regulation at the ionotropic GABA_A_R. Ionotropic activation depends on the intra-and extracellular concentrations of chloride, in turn regulated by the cation chloride cotransporter (CCC) family of membrane proteins. CCC are coupled with potassium (KCCs), sodium (NCCs) and both potassium and sodium (NKCCs). Both NKCC1 and KCC2 control reverse potentials to determine currents mediated by GABA_A_R and glycine receptors. During the first 1,000 days of brain development, NKCC1 facilitates excitatory activity on neuronal and glial precursor cell populations. As development proceeds, NKCC1 expression decreases and the expression of KCC2 leads to an inhibitory effect of GABA_A_Rs activation in the maturing brain.

Transition from fetal to neonatal life commonly triggers early NS, usually in association with fetal distress and neonatal depression which lowers the seizure threshold. Seizure occurrence within the first hours after birth is also moderated by the transient protective effect of maternal oxytocin released during labor that drives the chloride transporter balance toward greater inhibition (195) by temporally activating KKC2 control which suppresses NS. Seizure-onset signifies a return to the pre-labor abnormal excitatory action of NKCC1 related to the underlying etiology and timing of brain injury, either close to or independent labor and delivery.

**Figure 7:**
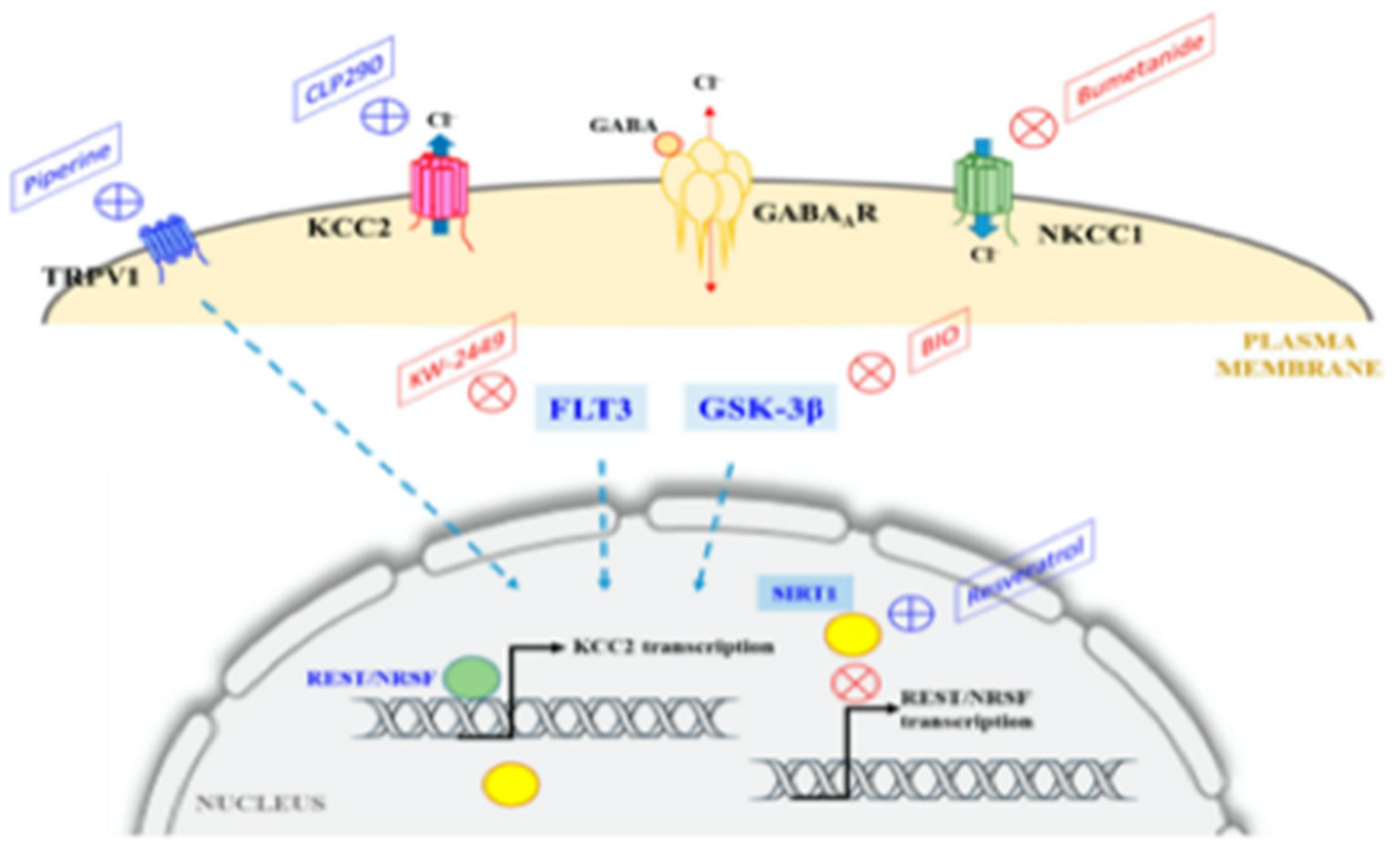
A schematic diagram illustrating the major chloride transporters KCC2 and NKCC1, whose activities determine intracellular Cl—concentration and EGABA, and the pharmacological manipulations that could potentially correct for a loss of KCC2 levels or activity under neuropathological conditions (where GABAergic transmission could become excitatory instead of inhibitory). The classical NKCC1 antagonist bumetanide has been shown to be efficacious in this regard. As discussed in the text, CLP290 enhances the plasma membrane expression of KCC2, while compounds that target TRPV1, FLT3, or GSK-3 β also modulates KCC2 expression (dotted arrows), but the mechanisms involved are unclear. Resveratrol activation of SIRT1 could downregulate the expression of RE1-silencing transcription factor/Neuron-restrictive silencer factor (REST/NRSF), which suppresses KCC2 expression (197).

Clinical or electrographic seizure-onset may be independent of or in addition to intrapartum events (158), with or without fetal distress. Early seizures shortly after delivery may signify predominately fetal brain lesions from antepartum diseases (157). Future investigations are needed to explore the relationships between prenatal and postnatal factors that result in imbalance between KKC2 and NKCC1, facilitating NS. Therapeutic strategies that modify chloride transporter effects to treat NS were investigated using bumetamide, a diuretic that antagonizes NKCC1 cotransporters. Although this particular off-label drug use failed efficacy criteria, other pre-clinical trials of similar analogs are being studied as alternative experimental options to investigate (196) (Figure 7).

### Neonatal Stroke Syndromes

A recent population-based study reported NSK prevalence of 1:1,100, with higher estimates for male neonates (198). AIS accounts for 80% of this cohort, with the remainder presenting as venous thrombotic or hemorrhagic strokes. NSK can present as NE ± NS after fetal distress and neonatal depression. NS without NE may be the initial clinical sign that initiates the diagnostic process revealing NSK. Neonatal medical illnesses associated with infections, coagulopathies and severe volume depletion can result in postnatal NSK. These conditions, non-etheless, may be associated with MPF triad diseases. Antepartum and peripartum etiologies associated with NSK include maternal infections associated with chorioamnionitis/funisitis, fetal malperfusion syndromes, or maternal/fetal coagulopathies from genetic or acquired diseases that contribute to hepatobiliary disease after HI/inflammation. These diseases include mechanisms that promote thrombotic or embolic forms of NSK. Systemic diseases may worsen with disseminated intravascular coagulation before or after birth, associated with severe HI or infection. Intracranial hemorrhage in the preterm brain may originate in the germinal matrix, with progressive worsening grades of intraventricular hemorrhage, culminating in hemorrhagic venous stroke within brain parenchyma. Stroke syndromes may later present as childhood motor asymmetries or symptomatic seizures, having escaped detection as MPF triad disorders or NSK.

A recent consensus statement merge genetic and acquired factors as responsible for arterial ischemic (AIS), venous thrombotic and hemorrhagic presentations of childhood stroke (199), acknowledging knowledge gaps at the time of diagnosis. Neonatal inflammatory markers have been reported, suggesting prenatal disease-risk for NSK (200). However, most studies have not comprehensively studied trimester-specific MPF triad factors that could potentially be associated with NSK (201). Less than a quarter of reported NSK cases included postnatal placental/cord analyses (202), which could offer a more specific timeline when a disease process began and evolved.

NSK are associated with trimester-specific MPF triad factors, beginning with developmental anomalies affecting early cerebrovasculature (203). Genetic and epigenetic alterations in signaling pathways impair vasculogenesis and later angiogenesis, altering the development of arterial and venous vascular structures. Abnormal cerebrovascular development then contribute to third-trimester diseases from HI and inflammation, affecting coagulation, and hemostatic pathways resulting in thrombotic or embolic sources of stroke (201). Antepartum or peripartum onsets depend on the type and severity of congenital or acquired cerebrovascular diseases, placental/cord vasculopathies, and systemic diseases involving coagulation and hemostasis. Paradoxical emboli can cross into the fetal systemic circulation through a patent foramen ovale or the ductus venosus, with or without anomalous access because of CHD. Venous forms of NSK reflect systemic hemostatic disorders, associated with acquired infections, inherited blood dyscrasias, or severe intravascular depletion. Both arterial and venous NSK can convert from ischemic to hemorrhagic pathophysiologic expressions, with the latter associated with greater complications from cerebral edema and herniation syndromes. Focal or multifocal injuries follow arterial or venous vascular distributions, but also may mimic or accompany HI patterns of injury on neuroimaging. Placental/cord findings such as fetal thrombotic vasculopathy and umbilical vein thrombosis exemplify embolic sources for arterial NSK from MPF triad diseases with or without the association with the GOS.

### Encephalopathy of Prematurity

The preterm population is the largest group representing the GNNS, with a global estimate of 10.6% in 2014. Significant racial and ethnic disparities in developed and developing countries (204) explain significant variations in incidence. The global burden of preterm birth needs to be addressed to achieve sustainable development goals to reduce morbidities and mortalities (9). The neonate less than 37 weeks defines prematurity, with 70% represented by late preterm infants between 34 0/7 and 36 6/7 weeks. Given the limits of accuracy to ascertain gestational age (GA) by at least 2 weeks, early term neonates may functionally express preterm organ-specific development, with greater morbidities based on physiologic vulnerabilities. As GA approaches the lower limits of viability, each organ system expresses more significant immaturity of structure and function, increasing susceptibilities to diseases or adverse conditions. Expressions of dysmaturity or injury by preterm neonates differ among organs. Trimester-specific MPF triad conditions result in altered fetal brain development, superimposed on postnatal changes for those born as preterm neonates.

Birth cohorts exemplified by the INTERGROWTH 21st Project describe the distribution of clinical phenotypes associated with the preterm birth syndrome (205), representing one category of the GOS. This study of over 60,000 maternal-child pairs offered fetal and neonatal anthropometry and classified 12 preterm birth clusters according to different MPF triad risk factors. Many of these factors could be associated with early or late pregnancy effects, as previously discussed, applicable to MIA and the GOS as representative pathophysiologic pathways. The largest single cluster, however, consisted of 30% without identified MPF conditions. None of the 12 clusters for this entire cohort included placental/cord or genetic datasets. These data might have identified additional associations, particularly within the subset with no identifable conditions. Subsequent birth cohort studies have included placental/cord (206, 207) and genetic analyses, identifying G × E interactions associated with preterm phenotypes. Genetic loss or gain of function contributes to premature birth associated with the GOS (208, 209). Organ-specific diseases of prematurity also have genetic influences, including intraventricular hemorrhage (210), retinopathy of prematurity, chronic lung disease, and necrotizing entercolitis (211). Placental/cord genetic (84, 212, 213) and epigenetic (214, 215) studies further underscore the relevance of genetic resilience or vulnerability for neurologic sequalae based on shared parental/fetal genetic material in the placenta culminating in premature births.

EP refers to the combined expression of developmental and destructive influences on the maturing nervous system (216) in response to multi-systemic diseases and conditions from prenatal to postnatal life. Healthy preterm survivors have a greater likelihood for functionally normal outcome. However, even with this healthy preterm cohort, survivors will demonstrate structural and neurophysiological expressions of EP dependent on the degree of GA immaturity. MPF triad and neonatal factors associated with conditions of prematurity without overt diseases may therefore carry a higher risk for sub-optimal neurodevelopmental outcome, particularly as GA approaches the lower limits of viability. This risk increases with organ-specific disease effects on brain development, such as encephalopathy of congenital heart disease (217). With multiple organ illnesses, there is a greater risk for altered long-term health outcomes to multiple systems affecting NS development (218). Compromised outcome particularly worsens below 28 weeks GA, defined as the extremely preterm neonatal (EPT) survivor. While decreasing rates of cerebral palsy rates are now reported for the EPT cohort, concerns for cognition, behavior, and mental health disorders remain (219). Poor academic performance (220) and disorders of social competence (221, 222) expressed by the EPT cohort are childhood performance markers for reduced quality of life and employment potential as well as mental disorders (223) during adulthood (224, 225).

Pre-conceptional toxic stress to the mother and intrauterine stresses to the MPF triad adversely alter fetal brain development and contribute to preterm birth as one of the GOS. Maternal adversities resulting from mental health illnesses, socioeconomic disparities, and abusive situations highlight how altered neurogenesis include G × E interactions that begin before conception and worsen with ongoing toxic stress during pregnancy (226), affecting fetal brain development. Postnatal adversities contribute to this prenatal neuronal dysgenesis, with slower and more atypical extrauterine neurogenesis (227). Medical interventions for conditions of prematurity such as respiratory and fluid/nutritional treatments introduce pain and discomfort despite the delivery of high-quality clinical care. Altered hypothalamic–pituitary axis stress responses from these interventions influence the extent and complexity of brain connectivities, specific to vulnerable brain regions such as the CA1 and CA3 regions of the hippocampus (228–230).

Neuroimaging, neurophysiological and neurobehavioral differences have been described in healthy populations as early as corrected term GA when compared with full-term controls (173, 231, 232). Altered volumetric measurements and functional MRI patterns, even without demonstrable diseases or injuries in healthy preterm cohorts are correlated with lower psychometric scores at preadolescent and adolescent school ages. More significant cognitive/behavioral morbidities such as learning disabilities, disorders of attention and social competence may result as more severe medical illnesses are experienced by the MPF triad and the neonate. However, complex G × E interactions increase or lessen sequalae severity based on genetic vulnerability or resilience. More compromised outcomes such as severe intellectual disabilities and autistic spectrum may nonetheless be expressed despite minimal medical illnesses to the MPF triad or preterm survivor based on more pervasive G × E interactions earlier after conception.

## A Fetal/Neonatal Neurology Program Includes Convalescent Neonatal Care

Prenatal consultative involvement by the FNN facilitates convalescent medical management of the neonate following the acute phases of the GNNS as the family assumes a greater role in daily care. Serial clinical assessments are integrated with placental analysis, neuroimaging, and genetic testing to formulate a more comprehensive horizontal/vertical diagnostic profile of the child that can be communicated to the family. Important clinical perspectives are required by nursing and therapists' assessments to appropriately prepare the child for transition to home.

Multiple interdisciplinary developmental care interventions for families are being successively reassessed by nursing and neonatal therapy researchers (233–235). Design of these studies requires consideration of improvements in neonatal unit construction (236) that encourage family participation. Improved outcomes are being reported for specific groups of neonatal survivors, validated by environmental enrichment studies that apply relevant developmental neuroscience principles to animal (237) and human cohorts (238). Preterm survivors who require NICU care for the longest times have been the most frequently studied cohort given their greater risks for neurologic deficits from EP. Non-pharmacologic treatments range from easily administered skin-to-skin care to more sophisticated multi-sensory interventions (e.g., NIDCAP) requiring advanced training to administer. While earlier and more aggressive interventions are generally advocated, few comparative studies differentiate which neonatal phenotypes optimally respond to a particular intervention (239). Variations in treatment efficacy are based on differences in G × E interactions for each neonate. Response differences may reflect MPF triad conditions, gestational maturity, type/severity of organ-specific diseases or regularity and intensity of the chosen intervention. Continuity of benefits from different developmental care interventions for defined preterm-risk groups after discharge also has not been systematically studied relative to family resources, parental compliance, or obstacles to medical care. These variables influence neurodevelopmental improvements during critical/sensitive periods of brain maturation over the first 1,000 days, prior to as well as after enrollment in a multidisciplinary early intervention program.

## Outpatient Pediatric Care Supported by the Fetal/Neonatal Neurology Program

Multiple interdisciplinary conferences with the family offer opportunities to answer questions and discuss the diagnosis, prognosis, and therapeutic plans prior to discharge. A detailed medical summary is then provided to the primary care practitioner, with an early outpatient appointment scheduled to maintain continuity of care. The family should be introduced to a designated FNNP team consisting of a neurologist, developmental/behavioral specialist, nurse practitioner, and relevant therapists. Optimal communication needs to be maintained between as well as at each outpatient assessment.

Developmental disorders and epilepsies expressed over the first 2 years of life need to be addressed in the outpatient clinic or the hospital/ICU setting. Pediatric presentations of neurologic conditions may be the first opportunity to evaluate the child who experienced prenatal brain disorders without expression of the GNNS. Involvement by the FNN in a high-risk MFM service helps identify survivors even without formal prenatal consultations with the family. The pediatric neurologist may first meet the family during the convalescent stage of neonatal care in the step-down nursery. Neonatal survivors who experienced the GOS and GNNS alternatively are more likely to require earlier medical interventions following discharge, including pediatric neurointensive care (240). Neonatal critical care survivors disproportionately represent multiple pediatric ICU admissions (241, 242). Strategies to develop pediatric neurocritical programs must consider high-risk neonatal survivors (243) beginning during the first 1,000 days. Medically fragile children are more likely to suffer more severe intercurrent illnesses (244) with advancing ages into childhood. Children with failure to thrive, CHD, and other complex multi-organ disorders from genetic or acquired diseases exemplify cohorts who more likely will require medical attention including hospitalizations. Acute and non-acute pediatric neurology consultations will be requested by multiple pediatric subspecialties if communicable and non-communicable diseases adversely affect or worsen neurologic function as a result of organ-specific systemic diseases.

## Later Childhood and Adolescent Involvement

Earlier involvement during the first 1,000 days facilitates continued pediatric neurology consultative input throughout childhood to more effectively address ongoing or new medical illnesses affecting neurologic/behavioral performance. Children may present during their school years requiring coordination with educators to optimize their individual educational plans. Accompanying behavioral disorders such as attention hyperactivity disorders and sleep disorders will require evaluation and possible pharmacologic interventions. Neuropsychological evaluations may need to supplement school evaluations for children with more challenging cognitive/behavioral deficits. Coordination of care with mental health providers may need to address multiple forms of anxiety, mood, and psychotic disorders, particularly during maturation into adolescence. Neurologic disorders may present at older ages after the child experiences acute infectious or inflammatory illnesses, craniocerebral trauma, worsening systemic diseases, or adverse environmental exposures which adversely alter developmental neuroplasticity processes as explained by OA. Transition of neurologic care into adulthood presents new challenges for those expressing developmental disorders, intellectual disabilities, and pharmaco-resistant epilepsies. Communication by the pediatric neurologist to the adult neurologist or other adult medicine primary care providers will extend the depth of diagnostic, therapeutic, and prognostic insights of disease processes that initiated during the first 1,000 days.

## Research Advances Relevant to the First 1,000 Days

Neuroinformatics is one research discipline that will improve diagnostic accuracy during the first 1,000 days. Optimal data analyses using a more complete “first 1,000 day” informatics platform can more accurately address multifactorial statistical analyses (245) of the MPF triad, neonate, and child. A comprehensive and flexible relational database will enhance future clinical service, educational curricula for trainees, and research efforts for collaborating faculty at academic centers. Multi-center studies increase statistical power using larger population sizes across diverse socioeconomic, ethnic, and racial backgrounds. More comprehensive educational curricula that integrate trimester-specific MPF triad, neonatal, and childhood factors will use these platforms to enhance the diagnostic skills for the next generation of multidisciplinary healthcare providers (246). Collaboration among multiple national and international academic centers will more accurately follow outcomes to develop more effective medical interventions and advocate for medical equity regarding healthcare policy.

Multi-modal diagnostic protocols represent another research area that will advance the diagnostic process, beginning during the first 1,000 days. Future applications of placenta/genetic research findings will enhance trimester-specific MPF triad considerations. Placental cell culture, omics, imaging, and genetic biomarkers include an extensive list of technologies that can document structural and functional alterations of the placenta and cord (85) during pregnancy. Correlations with postnatal histopathology and clinical outcome will yield useful insights. G × E interactions involving post-mitotic modifications such as telomeric shortening (247) from MPF triad and neonatal diseases or conditions will provide timelier diagnostic results for more effective therapeutic interventions. Multimodal fetal imaging protocols can compare fetal brain (248), placental and systemic organ (249) structural and functional interrelationships during development to assess health or disease of the fetal and neonatal brain applying a systems-biology perspective. Prenatal functional MRI of fetal brain development (250) can document region-specific variations in brain development relevant to postnatal learning and behavior. Neonatal quantitative brain MRI studies have assessed effects of medical interventions such as respiratory care (251) on brain region-specific functional and volumetric measures. Tissues and cell types profiled in the Roadmap Epigenetics Consortium (252) have identified human accelerated gene regions common to brain and non-CNS tissues. This genetic databank can be applied to longitudinal studies of healthy children compared with those who express neurologic/mental health disorders across maturation, beginning during the first 1,000 days.

## Neurotherapeutics to Improve Outcomes and Reduce Global Neurologic Burden

Timelier evaluations will promote neurotherapeutic research that improves outcomes across the lifespan. Earlier detection of fetal and neonatal brain disorders will promote the development of more effective neurotherapeutic interventions to prevent, rescue, and repair brain during critical/sensitive periods of brain development (1). Developmental neurotherapeutics will be offered during the first 1,000 days to those with pre-clinical vulnerability as well as others with early disease expression, selecting the appropriate treatment modality for optimal efficacy. By targeting specific sites at the synapse, neuron or circuit level (253), treatments will match specific experience-dependent neuroplasticity to address loss or gain of function from disease states such as hypoxia (254), applying epigenetic or omics biomarkers to assess neurologic recovery.

Reduction of neurologic disease burden across the lifespan should be one of the sustainable goals for maternal and pediatric health initiatives (255) adopted by individual nations and the global health community. A significant pediatric percentage contributes to the global burden of neurologic disease, as measured by disability-adjusted life years (256). Assessing the impact of medical interventions on both present and next generations regarding plasticity, growth, neurocognitive development and later development of non-communicable diseases at various stages of the life course will be a guide for public health prioritizations (257). Improvements in personalized patient care and healthcare policy define a nation's medical and economic health which in turn contributes to priorities in global health. Cooperation among government, industry, and non-profit organizations can collectively advocate for health policy for populations specific to developing and developed nations. Research on prenatal and early childhood brain disorders in the context of health disparities will more effectively reduce disease burden and financial costs. Interventions will be offered that are population and person-centered as well as gender-specific and sensitive to sexual orientation (258). Women's outcomes research will consider treatment efficacy both during and beyond the reproductive years. Neurotherapeutics offered during the first 1,000 days will contribute to improved scholastic success, employability, and quality of life that extend into adulthood.

## Developmental Origins and Life-Course Theories

The perspective that considers “the continuity of reproductive risk and the continuum of care-taking casualty” starts with growth and development during the first 1,000 days (7). Birth cohorts research continue to reinforce the importance of early-life factors that influence later-life health (259). The scientific disciplines of developmental origins of health and disease and life-course science have incorporated birth cohort findings into the more recent WHO Millennium sustainable goals such as the every newborn action plan (260).

**Figure 8:**
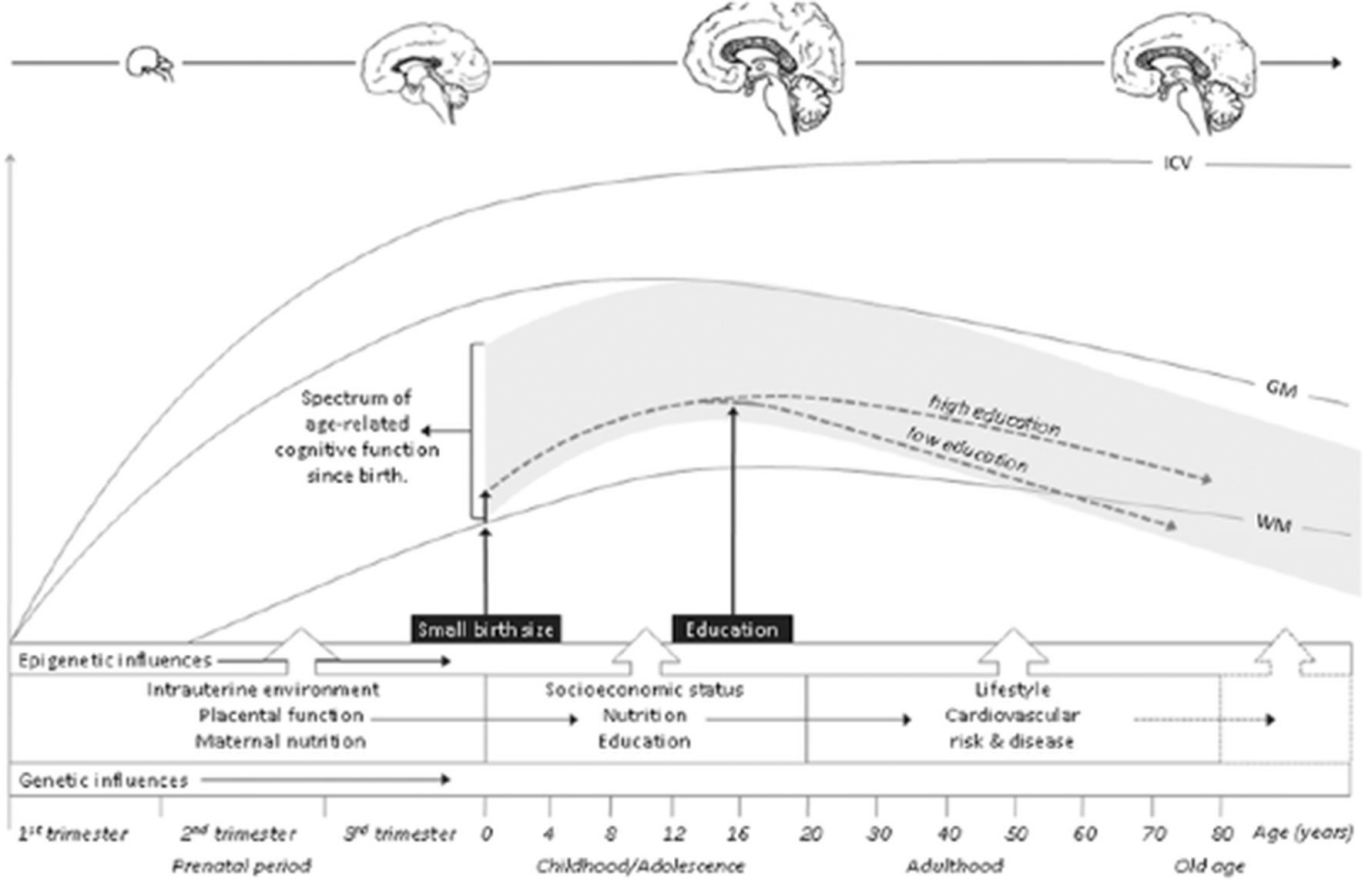
A hypothesized model of the origins and life course of brain aging. Several “critical periods” (prenatal period, childhood/adolescence, adulthood, and old age) are identified during which an individual is at greatest risk of damage if exposed to putative risk factors. Normal development of ICV and brain volumes (GM and WM) is presented for these critical periods, and the possible different risk factors influencing brain development throughout these periods are described. Allegedly, genetics and epigenetic influences could alter brain structure and function throughout life, but their impact would probably fade with age. In addition, the spectrum of age-related cognitive ability from birth to old age is presented in this figure, with a schematic view of our findings that small birth size is related to poor cognitive functioning only in those with lower educational levels (261).

Developmental origins and life-course theories stress the relevance of FNN when considering the diagnosis and treatment of adult-onset cerebrovascular, cognitive, neurodegenerative disorders and mental health disorders (1). One representative longitudinal study reported neurologic outcome to a mean age of 75 years (Figure 8) in relation to one of the GOS. Exposure to fetal growth restriction (i.e., defined as a reduced ponderal index) contributed to smaller brain volume by quantitative brain MRI studies and more rapid cognitive decline by neuropsychometric testing. While subsets of this cohort were subjected to the adverse effects of mid-life cardiovascular disease, diabetes and smoking, the limited sample sizes for these risk factors across the three groups of ponderal indices, only reduced educational opportunities reached statistical significance (261). Multiple birth cohort studies, however, have reported more significant correlations with these other factors, specifically related to the adverse consequences of the metabolic syndrome expressed as cardiovascular disease, diabetes, and obesity (262, 263). This approach should be the conceptual guidepost for future birth cohort studies that will support the importance of FNNP development, applied to the continuity of health care throughout the lifespan and across successive generations (257).

## Author Contributions

The author confirms being the sole contributor of this work and has approved it for publication.

## Conflict of Interest

The author declares that the research was conducted in the absence of any commercial or financial relationships that could be construed as a potential conflict of interest.

## Publisher's Note

All claims expressed in this article are solely those of the authors and do not necessarily represent those of their affiliated organizations, or those of the publisher, the editors and the reviewers. Any product that may be evaluated in this article, or claim that may be made by its manufacturer, is not guaranteed or endorsed by the publisher.
